# Phosphorylation of USP20 on Ser334 by IRAK1 promotes IL-1β-evoked signaling in vascular smooth muscle cells and vascular inflammation

**DOI:** 10.1016/j.jbc.2023.104911

**Published:** 2023-06-11

**Authors:** Lisheng Zhang, Jiao-Hui Wu, Pierre-Yves Jean-Charles, Pavitra Murali, Wenli Zhang, Aeva Jazic, Suneet Kaur, Igor Nepliouev, Jonathan A. Stiber, Kamie Snow, Neil J. Freedman, Sudha K. Shenoy

**Affiliations:** 1 Department of Medicine (Cardiology), Duke University Medical Center, Durham, North Carolina, USA; 2 Department of Cell Biology, Duke University Medical Center, Durham, North Carolina, USA

**Keywords:** de-ubiquitination, IRAK1, NFκB, TRAF6, vascular injury, interleukin-1β

## Abstract

Reversible lysine-63 (K63) polyubiquitination regulates proinflammatory signaling in vascular smooth muscle cells (SMCs) and plays an integral role in atherosclerosis. Ubiquitin-specific peptidase 20 (USP20) reduces NFκB activation triggered by proinflammatory stimuli, and USP20 activity attenuates atherosclerosis in mice. The association of USP20 with its substrates triggers deubiquitinase activity; this association is regulated by phosphorylation of USP20 on Ser334 (mouse) or Ser333 (human). USP20 Ser333 phosphorylation was greater in SMCs of atherosclerotic segments of human arteries as compared with nonatherosclerotic segments. To determine whether USP20 Ser334 phosphorylation regulates proinflammatory signaling, we created USP20-S334A mice using CRISPR/Cas9-mediated gene editing. USP20-S334A mice developed ∼50% less neointimal hyperplasia than congenic WT mice after carotid endothelial denudation. WT carotid SMCs showed substantial phosphorylation of USP20 Ser334, and WT carotids demonstrated greater NFκB activation, VCAM-1 expression, and SMC proliferation than USP20-S334A carotids. Concordantly, USP20-S334A primary SMCs *in vitro* proliferated and migrated less than WT SMCs in response to IL-1β. An active site ubiquitin probe bound to USP20-S334A and USP20-WT equivalently, but USP20-S334A associated more avidly with TRAF6 than USP20-WT. IL-1β induced less K63-linked polyubiquitination of TRAF6 and less downstream NFκB activity in USP20-S334A than in WT SMCs. Using *in vitro* phosphorylation with purified IRAK1 and siRNA-mediated gene silencing of IRAK1 in SMCs, we identified IRAK1 as a novel kinase for IL-1β–induced USP20 Ser334 phosphorylation. Our findings reveal novel mechanisms regulating IL-1β-induced proinflammatory signaling: by phosphorylating USP20 Ser334, IRAK1 diminishes the association of USP20 with TRAF6 and thus augments NFκB activation, SMC inflammation, and neointimal hyperplasia.

Activation of the proinflammatory transcription factor NFκB fundamentally involves lysine-63-linked, reversible polyubiquitination that activates signaling proteins constituting networks that converge to activate IκB kinase-β. These networks are engaged by pro-atherogenic (1, 2) cytokine receptors, including the tumor necrosis factor receptor-1 and Toll-like receptor-4 (TLR4)/interleukin-1 (IL-1) receptors, which activate ubiquitin E3 ligases of the TNFR-associated factor (TRAF) family (1). Downstream of TLR4/IL-1R signaling, TRAF6 is deubiquitinated (and thereby inactivated) by the deubiquitinase (DUB) known as ubiquitin-specific peptidase 20 (USP20) (3). Smooth muscle cell (SMC) USP20 activity suppresses vascular inflammation, as demonstrated in the context of carotid endothelial denudation in nonatherogenic mice (3) and atherosclerosis in *Ldlr*^−/−^ mice (4).

Ubiquitination-dependent signaling that culminates in NFκB activation is regulated, in part, by diverse DUBs that diminish activation of signaling protein networks. Furthermore, a particular DUB such as USP20 can regulate different substrates in NFκB-activating cascades. For example, USP20 deubiquitinates not only TRAF6 and β-arrestin2 to reduce TLR4-induced NFκB activation (3) but also RIPK1 to prevent TNFR-induced NFκB activation (4).

Because DUBs including USP20 are expressed as mature enzymes, the mechanisms by which a DUB binds to a substrate often defines the rate of substrate deubiquitination (5, 6). Furthermore, dynamic posttranslational modifications of a DUB, such as phosphorylation, acetylation, and SUMOylation affect its association with a substrate and thereby modulate the specificity and efficiency of substrate deubiquitination (7, 8, 9). Phosphorylation of USP20 on Ser334, for example, augments both the association of USP20 with the β_1_-adrenergic receptor and USP20’s deubiquitination of the β_1_-adrenergic receptor (10, 11). In contrast, phosphorylation of USP20 Ser334 reduces both the association of USP20 with the β_2_-adrenergic receptor and USP20’s deubiquitination of the β_2_-adrenergic receptor (10, 11). Nonetheless, the intrinsic DUB activity of USP20 is not changed by phosphorylation of USP20 on Ser334 (11).

To determine whether and how phosphorylation of USP20 on Ser334 affects TRAF6 deubiquitination and NFκB activation in SMCs, we employed gene-edited mice expressing a S334A mutant version of USP20.

## Results

### USP20 is phosphorylated on Ser333 in SMCs of atherosclerotic human arteries

**Figure 1:**
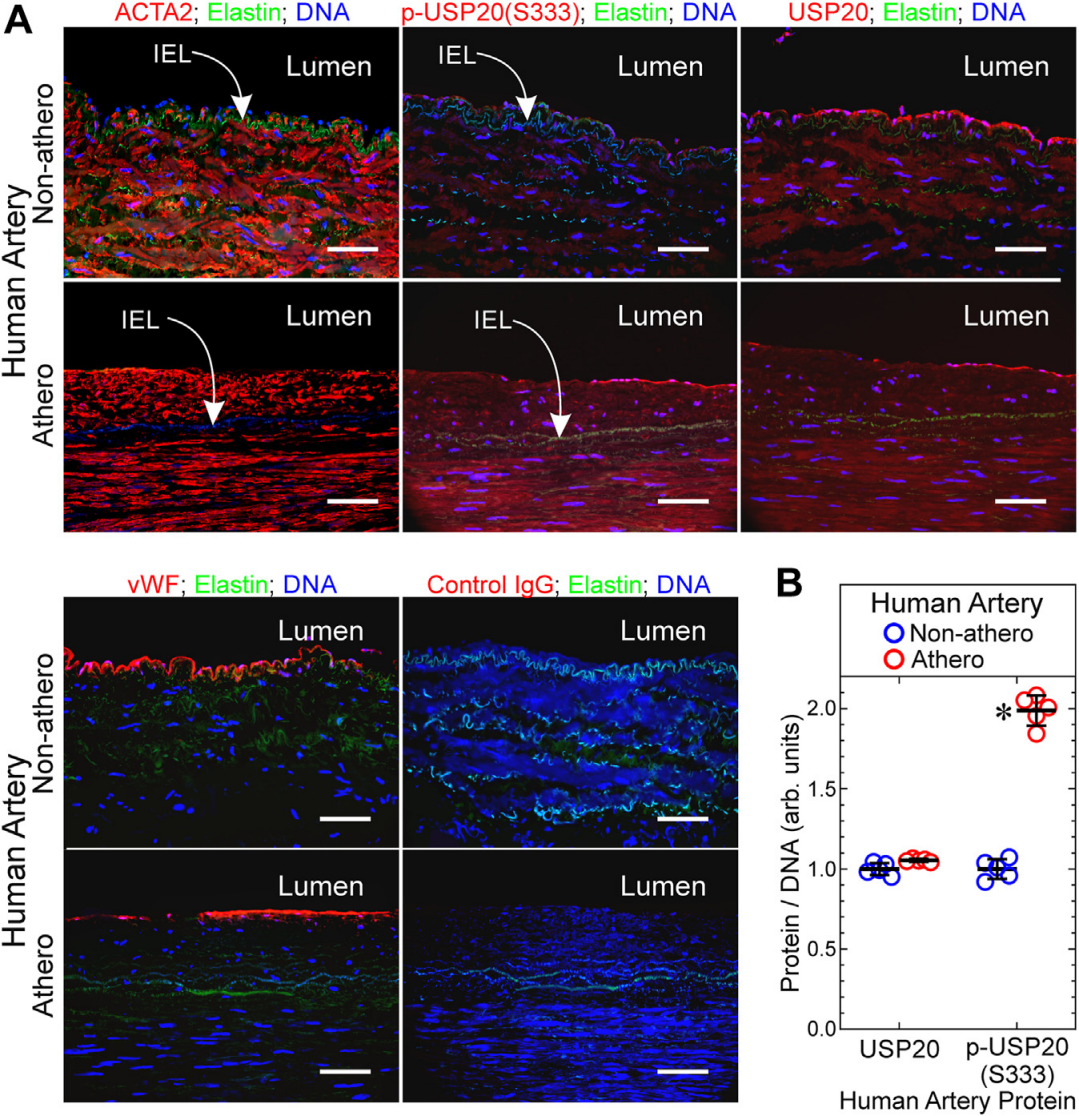
**USP20 is phosphorylated on Ser333 in SMCs and endothelial cells of atherosclerotic human arteries.** The peroneal arteries from amputated human legs were sectioned into atherosclerotic (“athero”) and relatively normal (“non-athero”) segments. *A*, serial sections were immunostained with Cy3-conjugated mouse IgG specific for smooth muscle α-actin (ACTA2, *top left*), Cy3-conjugated isotype control IgG (yielding no color, not shown); rabbit IgG specific for phospho-USP20(Ser333) (p-USP20), total USP20, the endothelial cell marker von Willebrand factor (vWF), or no particular protein (control IgG), and then anti-rabbit-Alexa 546 (*red*) and Hoechst 33342 (*blue*, DNA). Athero and nonathero sections were stained in parallel and imaged with identical camera settings; the FITC filter cube was used to visualize elastin autofluorescence (*green*). Scale bars = 100 μm. Shown are results from a single peroneal artery, representative of peroneal artery samples from five subjects. Lumen is oriented *upward*. *B*, for each artery, protein immunofluorescence intensity was normalized to DNA fluorescence within three randomly selected 20 × objective fields (encompassing ∼80% of the artery), by an observer blind to specimen identity. These ratios were then averaged for each specimen to obtain “Protein/DNA (arbitrary units)”, plotted as individual values and means ± SD for five distinct peroneal arteries. Compared with nonathero: ∗*p* < 0.05 (2-way ANOVA with Sidak test for multiple comparisons). IEL, internal elastic lamina; SMC, smooth muscle cell; USP20, ubiquitin-specific peptidase 20.

The USP20 seryl residue that is regulated by phosphorylation (10, 11) is conserved in both mouse (Ser334) and human (Ser333) USP20 orthologs. To discern whether USP20 Ser333 phosphorylation could be relevant in human atherosclerosis, we used IgG specific for phospho-USP20(Ser333) (10) to immunostain atherosclerotic human arteries obtained from legs that had been amputated because of arterial insufficiency (12). To minimize confounding by genetic differences among individual humans, we compared atherosclerotic with relatively nonatherosclerotic segments from individual arteries, as we reported (12). These atherosclerotic and nonatherosclerotic arterial segments demonstrated equivalent levels of total USP20 and smooth muscle α-actin (ACTA2) in the tunica media. However, the tunica media of atherosclerotic arterial segments demonstrated 1.9 ± 0.2-fold higher levels of phospho-USP20(Ser333) than the nonatherosclerotic arterial segments (Fig. 1). Thus, arterial inflammation inherent in atherosclerosis correlates with phosphorylation of USP20 on its regulatory residue Ser333.

### Preventing USP20 phosphorylation on Ser334 attenuates vascular inflammation

To investigate the role of USP20 Ser334 phosphorylation *in vivo*, we used a CRISPR/Cas9 (13) strategy to create a mouse expressing a USP20 mutant that harbors Ala instead of Ser at residue 334 (USP20-S334A, Fig. S1). The USP20-S334A mutant mimics unphosphorylated USP20 (10, 11). By cross-breeding S334A heterozygous mice, we obtained homozygous S334A mice and matching WT littermates to use for our studies. Mice homozygous for USP20-S334A displayed no overt phenotypic abnormalities. USP20-S334A and WT mice demonstrated equivalent weights (27 ± 2 *versus* 29 ± 2 gm respectively, for males and 21 ± 1 *versus* 22 ± 1, respectively, for females); systolic blood pressures (106 ± 6 and 109 ± 7 mm Hg, respectively; n ≥5 males per genotype); and heart rates (383 ± 14 and 397 ± 76 beats per minute, respectively; n ≥ 5 males per genotype). In addition, USP20-S334A and WT mice demonstrated equivalent USP20 protein levels in isolated SMCs (Fig. S2). USP20-S334A and WT mice also demonstrated equivalent mRNA levels of signaling components linked with vascular inflammation: TLR4, IL-1R, tumor necrosis factor receptor-1, MyD88, TAK1, and ASC (Fig. S3).

To determine whether SMC USP20 Ser334 phosphorylation modulates vascular inflammation *in vivo*, we subjected USP20-S334A and WT littermate mice to carotid endothelial denudation. By removing the endothelium, this wire-mediated procedure prompts neutrophils and platelets to adhere to the subendothelial extracellular matrix (14, 15). Subsequently, neutrophils and platelets secrete cytokines and growth factors that “activate” medial SMCs from their typically quiescent, contractile phenotype (14, 15). Activated SMCs proliferate and migrate across the internal elastic lamina into the subendothelial space, thereby creating “neointimal hyperplasia,” an inflammatory lesion that narrows the arterial lumen and thereby limits the efficacy of arterial stenting used to treat atherosclerotic arteries (14, 15, 16, 17, 18).

**Figure 2:**
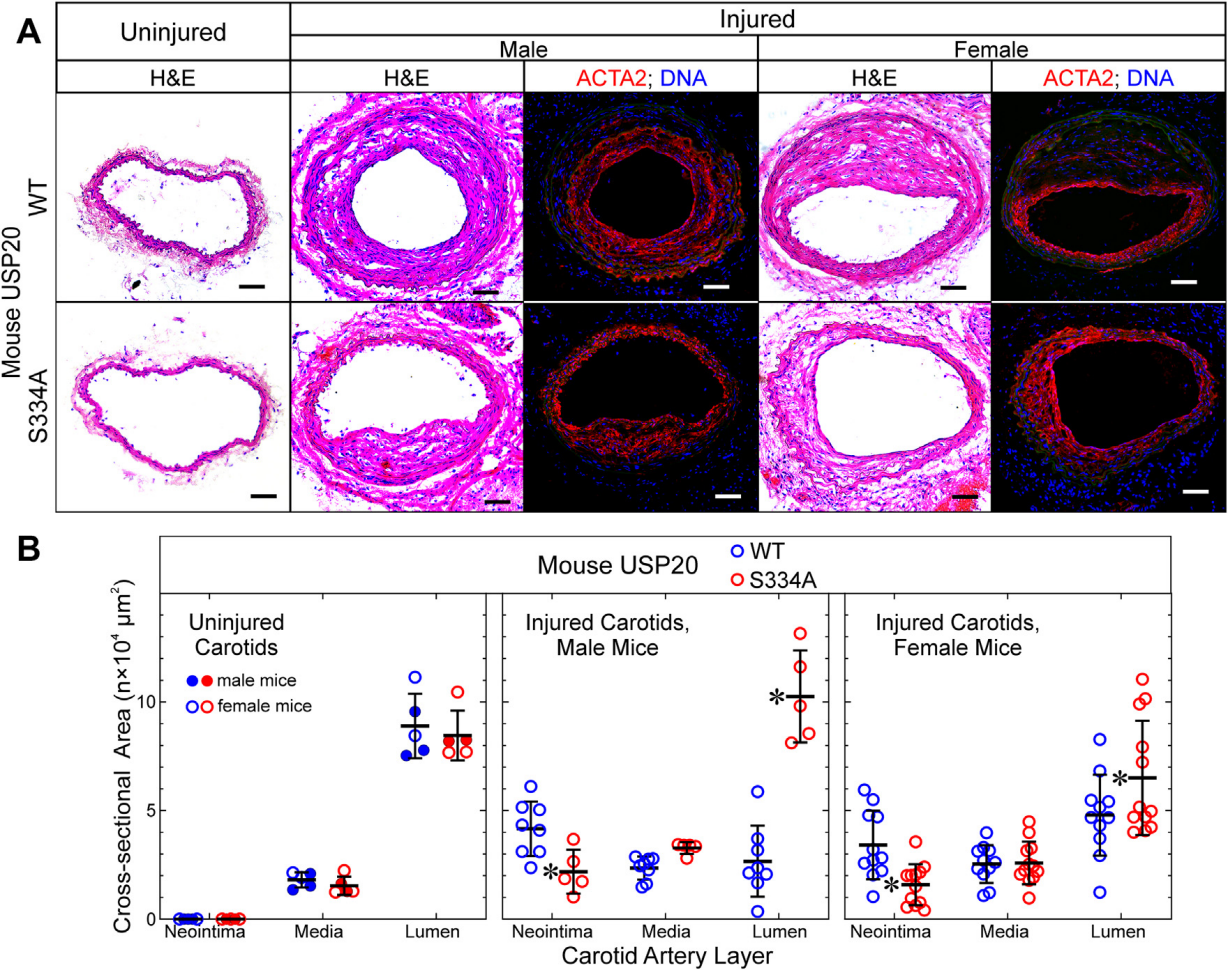
**Neointimal hyperplasia evoked by carotid endothelial denudation is exacerbated by phosphorylation of USP20-Ser334.** Female and male congenic mice of the indicated USP20 genotype were euthanized 4 weeks after wire-mediated carotid artery de-endothelialization. *A*, uninjured (*leftmost panels*) and contralateral de-endothelialized carotids (“injured,” eight panels) were embedded in Neg-50, frozen, sectioned at 5 μm, and stained with hematoxylin and eosin. Samples shown represent ≥5 mice of each genotype and sex. Scale bars = 50 μm. *B*, neointimal, medial, and luminal cross-sectional areas of uninjured carotids and injured carotids were measured by observers blinded to specimen identity. Mice were analyzed separately by sex, as indicated. Values were plotted, along with mean ± SD from ≥12 distinct female and ≥5 distinct male mice of each genotype. Compared with WT arteries by 2-way ANOVA with Sidak post-hoc test, ∗*p* ˂ 0.05. USP20, ubiquitin-specific peptidase 20.

Before endothelial denudation, carotid arteries of USP20-S334A and WT mice were morphologically equivalent (Fig. 2). However, 4 weeks after wire-mediated endothelial denudation, WT carotids demonstrated ∼2-fold greater neointimal hyperplasia than USP20-S334A carotids. Concordantly, USP20-S334A carotids had greater luminal areas than WT mice: ∼1.5-fold greater in females and ∼3-fold greater in males (Fig. 2). In this model of neointimal hyperplasia, the neointima comprises SMC-like (ACTA2^+^) cells (Fig. 2), as we have shown before (3, 12, 16). Thus, preserving a USP20 conformation that mimics its dephosphorylated state attenuates neointimal hyperplasia, which is triggered by arterial inflammation (16, 18, 19, 20).

**Figure 3:**
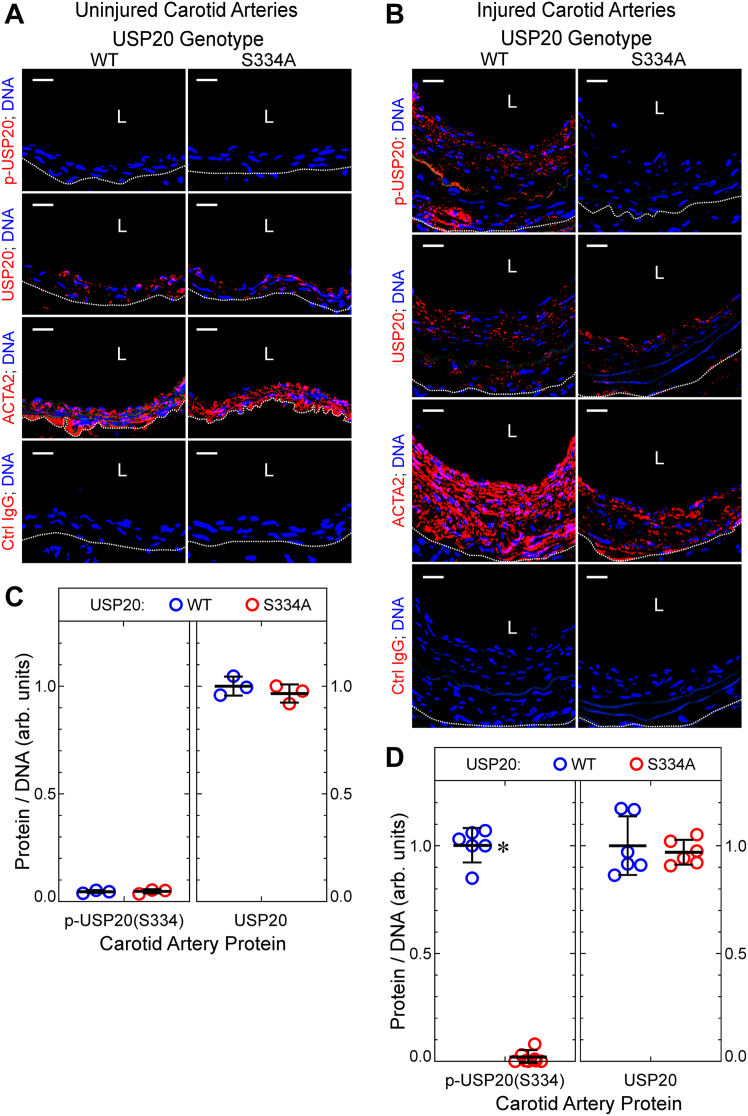
**Neointimal hyperplasia triggers phosphorylation of UPS20 on Ser334.** Carotid arteries of female mice from Figure 2 were serially sectioned and immunostained with IgGs specific for USP20 phosphorylated on Ser334 (“p-USP20”), total USP20, ACTA2, or no protein (isotype control, “Ctrl”), and counterstained for nuclear DNA (Hoechst 33342). Confocal fluorescence photomicrographs are shown from uninjured (native) carotids (*A*) and contralateral carotids subjected to endothelial denudation (“injured,” *B*); scale bars = 20 μm. *Dotted lines* denote the external elastic lamina. *C* and *D*, for each carotid artery section, protein immunofluorescence in the tunica media and intima was normalized to DNA fluorescence. These ratios were normalized to the mean value obtained for WT carotid arteries and plotted for individual mice (along with means ± SD) from three native carotids (*C*) and six injured carotids (*D*) from each genetic group. Compared with WT: ∗*p* < 0.01. L, lumen; USP20, ubiquitin-specific peptidase 20.

Did phosphorylation of USP20 on Ser334 occur during neointimal hyperplasia? We addressed this question by immunostaining carotid arteries with the same anti-phospho-USP20(Ser333) IgG used on human arteries in Figure 1, because the IgG also recognizes mouse USP20 phosphorylated on Ser334. Phosphorylation of USP20 on Ser334 could not be detected in mouse carotid arteries prior to endothelial denudation—even though USP20 itself was easily detectable (Fig. 3, *A* and *C)*. However, in the context of arterial inflammation associated with neointimal hyperplasia, phosphorylation of USP20 on Ser334 clearly manifested (Fig. 3, *B* and *D*). As expected (10, 11), no phosphorylation of USP20 on Ser334 was detectable in either native or injured arteries of the USP20-S334A mouse (Fig. 3). On the other hand, both S334A and WT arteries expressed equivalent levels of total USP20 in both native and injured arteries (Fig. 3). Thus, phosphorylation of USP20 on Ser334 occurs during atherogenesis in human arteries and during nonatherosclerotic neointimal hyperplasia in mouse arteries.

**Figure 4:**
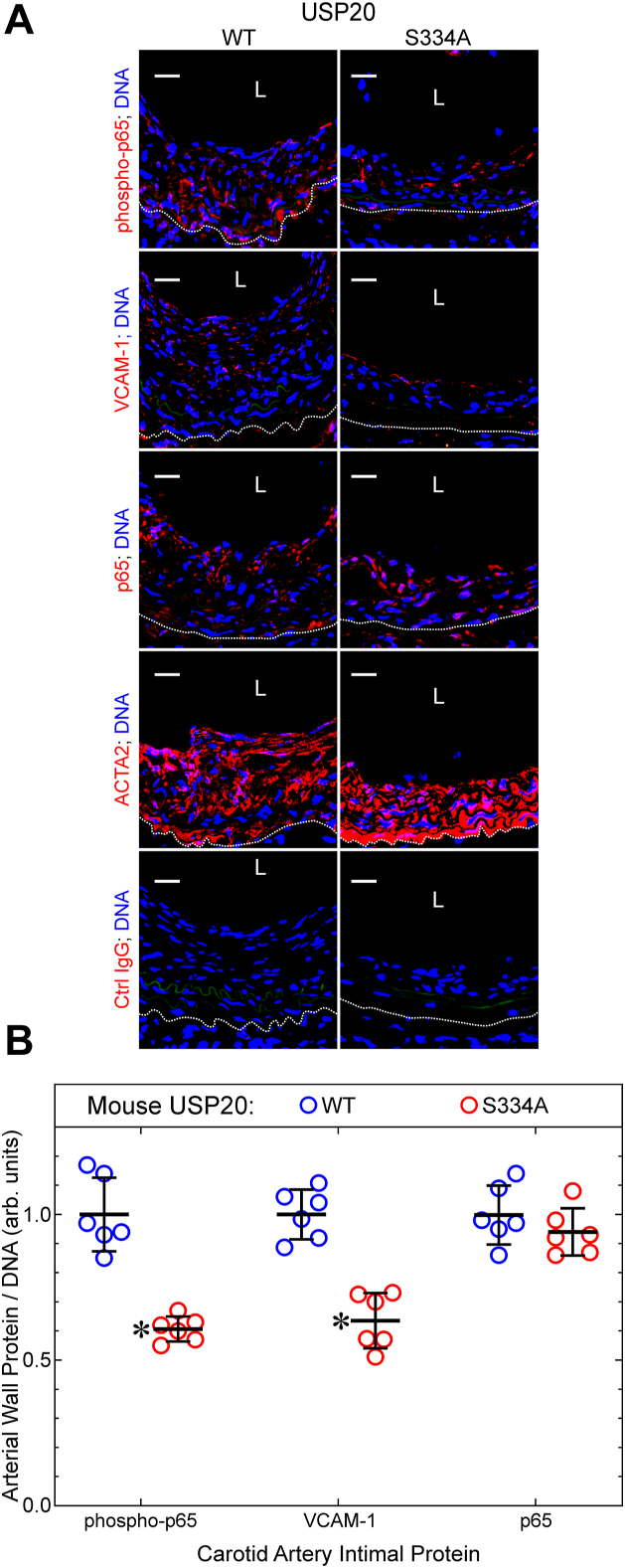
**Phosphorylation of USP20 on Ser334 augments arterial inflammation.***A*, carotid arteries of female mice from Figure 2 were serially sectioned and immunostained with IgGs specific for the p65 NFκB subunit phosphorylated on Ser536 (“phospho-p65”), the NFκB-dependent gene product VCAM-1, total p65, ACTA2, or no protein (isotype control (“Ctrl”) IgG). Each section was counterstained with Hoechst 33342 for nuclear DNA. Confocal fluorescence photomicrographs are shown; scale bars = 20 μm. The *dotted line* denotes the external elastic lamina. *B*, for each carotid artery section, protein immunofluorescence in the tunica media and intima was normalized to DNA fluorescence; these ratios were normalized to the average ratio obtained for WT carotid arteries and plotted as mean ± SD of six specimens per genetic group. Compared with WT: ∗*p* < 10^−4^ (2-way ANOVA with Sidak multiple comparisons test). L, lumen; USP20, ubiquitin-specific peptidase 20.

To determine whether USP20-S334A reduces arterial inflammation triggered by endothelial denudation, we examined carotid arteries for activation of the proinflammatory transcription factor NFκB, by two read-outs (19): (a) phosphorylation of the p65 NFκB subunit on Ser536—phosphorylation that is effected by IκB kinase-β (among other kinases), and that augments NFκB transcriptional activity (21, 22, 23); (b) expression of VCAM-1 (CD106), an integrin-binding protein that facilitates adhesion of monocytes and lymphocytes, and that is encoded by an NFκB-dependent gene (18, 24). By these read-outs, NFκB activation was ∼60% greater in WT than in USP20-S334A mouse carotids after endothelial denudation (Fig. 4). Levels of phospho-p65(Ser536) and VCAM-1 were concordantly 35 to 40% lower in USP20-S334A carotids than in WT carotids, even though protein levels of the NFκB p65 subunit were equivalent in WT and USP20-S334A carotids (Fig. 4). These data suggest that preventing USP20 Ser334 phosphorylation reduces NFκB activation in the context of neointimal hyperplasia.

Because neointimal hyperplasia integrally involves SMC proliferation (16), we asked whether USP20-S334A reduced SMC proliferation in the injured carotid arteries. Co-localization of the S phase marker PCNA with the SMC marker ACTA2 revealed that SMC proliferation was 38% less in USP20-S334A than in WT carotids 4 weeks after endothelial denudation (Fig. S4). Because net SMC hyperplasia reflects the balance of proliferation and apoptosis, we also examined carotid arteries for levels of activated or “cleaved” caspase-3, a marker of apoptotic cells. The prevalence of apoptotic cells in the arterial wall was 34% less in USP20-S334A than in WT carotids (Fig. S4). Lastly, because neointimal hyperplasia also comprises extracellular matrix, we assessed collagen I density and found it to be equivalent in USP20-S334A and WT carotids 4 weeks after endothelial denudation (Fig. S4). Altogether, these findings and Figure 4 suggest that phosphorylation of USP20 on Ser334 augments neointimal hyperplasia primarily by increasing inflammatory signaling and associated proliferation of SMCs.

**Figure 5:**
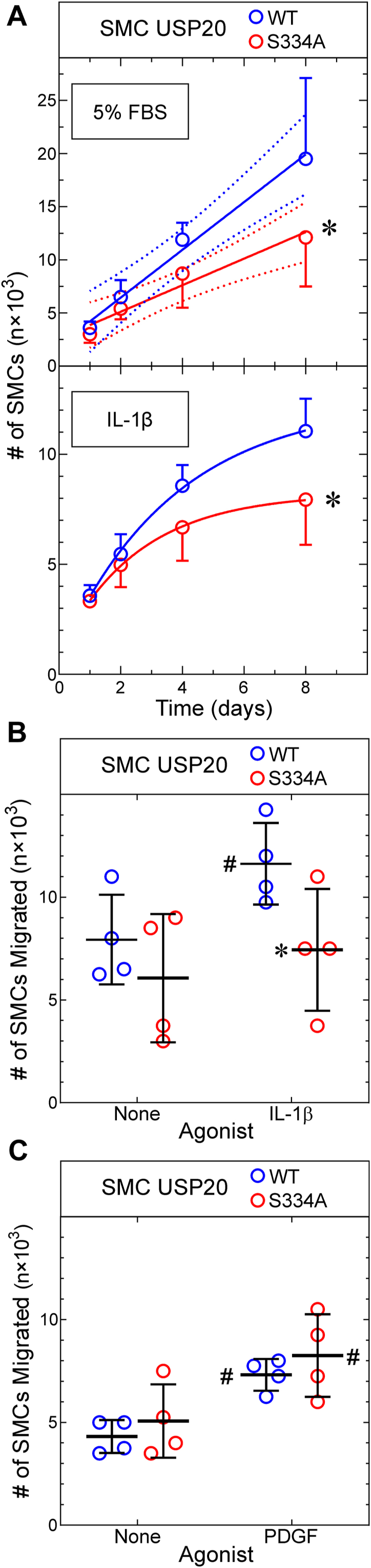
**Phosphorylation of USP20 on Ser334 augments SMC proliferation and migration.***A*, SMCs from WT and USP20-S334A mice were plated at 3 × 10^3^ SMCs/well in 96-well plates and stimulated to proliferate with either 5% FBS (v/v) (*top panel*) or IL-1β (20 ng/ml, *lower panel*), as indicated. Plates were harvested at the indicated times, and SMCs were quantitated as described in Experimental procedures. Shown are means ± SD from three independently isolated SMC lines of each genotype. FBS-stimulated growth was analyzed by linear regression (solid lines, R^2^ = 0.75 and 0.62 for WT and USP20-S334A, respectively). The line slopes were different from 0 (*p* ≤ 10^−4^); the 95% confidence limits for each line are displayed in *dotted lines* (*top panel*). Doubling times calculated from linear regression were 2.8 and 4.1 days for WT and USP20-S334A SMCs, respectively (starting at 2 days). IL-β-stimulated growth was fitted by nonlinear regression to an exponential plateau model, and curves were compared by the extra sum-of-squares F test. Compared with WT SMCs: ∗*p* < 0.03. *B* and *C*, SMCs from WT and USP20-S334A mice were plated on Transwell membranes (5 × 10^4^ SMCs/membrane); migration was evoked with 20 ng/ml of IL-1β (*B*) or 25 ng/ml PDGF-BB (*C*) as described in Experimental procedures. The number of SMCs migrated to the *bottom* surface of the membrane is plotted for four experiments with independently isolated SMCs of each genotype, along with means ± SD. Compared with cognate unstimulated SMCs: #*p* < 0.006; compared with WT: ∗*p* < 0.025 (paired *t* tests). IL-1, interleukin-1; SMC, smooth muscle cell; USP20, ubiquitin-specific peptidase 20.

To model neointimal hyperplasia *in vitro* (16, 17), we assessed proliferation and migration of SMCs derived from WT and USP20-S334A mice. Proliferation in response to 5% fetal bovine serum was ∼40% slower in USP20-S334A SMCs than in WT SMCs, as judged by SMC doubling time (Fig. 5*A*). We examined the effects of IL-1β on SMCs because of the importance of IL-1β in the pathogenesis of neointimal hyperplasia (25, 26, 27). IL-1β promoted considerably less proliferation than 5% FBS, and IL-1β promoted ∼34% less proliferation in USP20-S334A SMCs than in WT SMCs (Fig. 5*A*, bottom). Similarly, IL-1β induced migration in WT SMCs, but not in USP20-S334A SMCs (Fig. 5*B*). To determine whether this deficiency of USP20-S334A SMC migration was specific to IL-1β, we tested the SMC migration response evoked by PDGF-BB—because PDGF receptor signaling does not involve USP20. PDGF-BB induced equivalent migration in WT and USP20-S334A SMCs, even though the fold/basal migration evoked by PDGF-BB and IL-1β were comparable (1.7- and 1.5-fold, respectively, Fig. 5, *B* and *C*). Thus, USP20-S334A SMCs do not migrate normally in response to signaling triggered by IL-1β through the USP20 substrate TRAF6, but USP20-S334A SMCs do migrate normally in response to signaling that does not involve USP20 substrates. Taken together, these data suggest that phosphorylation of USP20 on Ser334 augments SMC proliferation and migration, particularly to agonists distinct from PDGF, like IL-1β.

### Preventing USP20 phosphorylation on Ser334 augments association with and deubiquitination of TRAF6

To understand mechanisms by which phosphorylation of USP20 on Ser334 may regulate SMC and vascular inflammation, we first asked whether USP20-S334A retains a WT level of ubiquitin-binding activity—because substrate binding is the rate-limiting step in DUB activity (6). To address this question, we used an active site–directed probe known as ubiquitin vinyl methyl ester (Ub-VME), a ubiquitin derivative that binds irreversibly to the catalytic site of active DUBs and thereby produces DUB-Ub-VME adducts (28). These adducts have a slower electrophoretic mobility than their cognate native DUBs. If the active site of a DUB is misfolded, the DUB will not bind Ub-VME.

**Figure 6:**
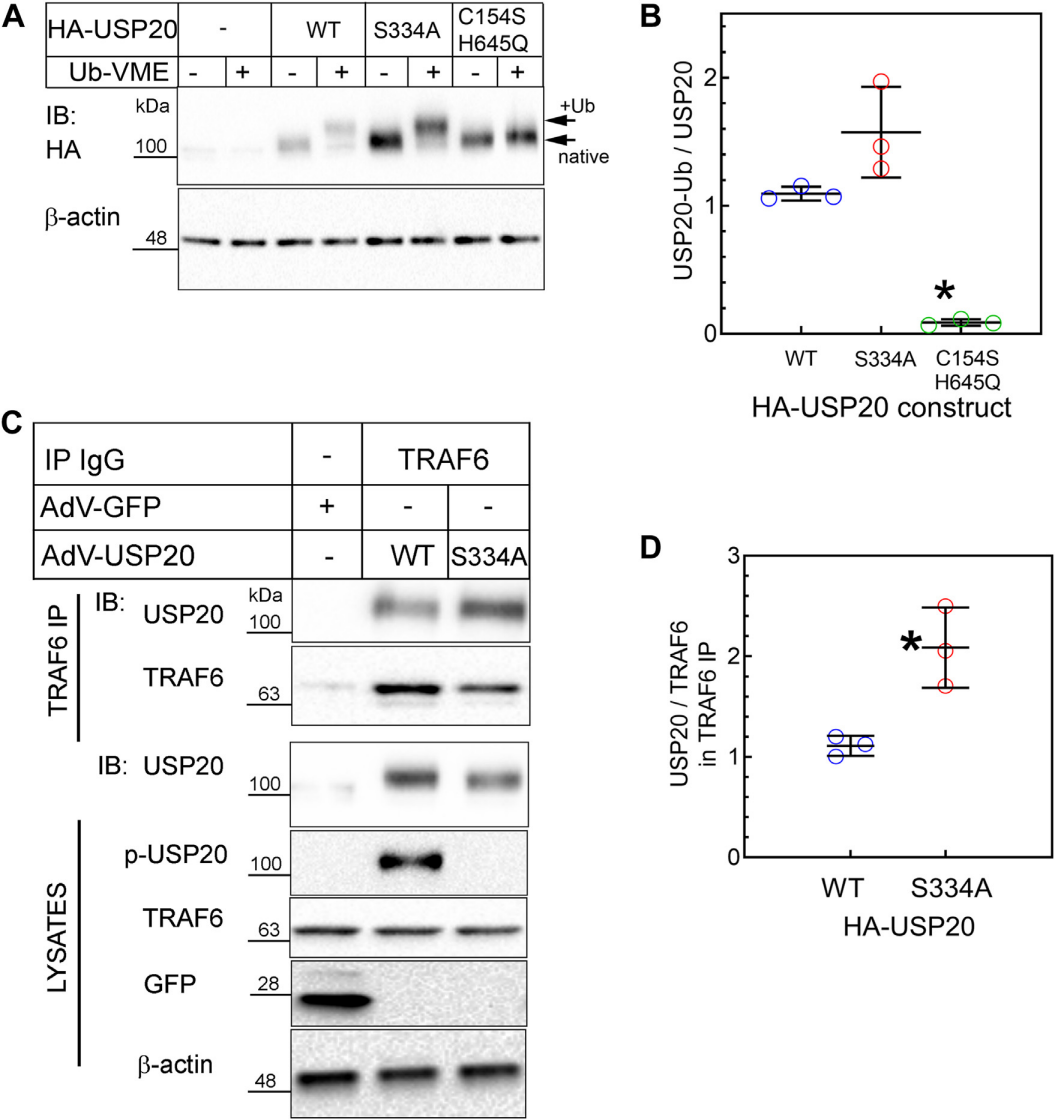
**Phosphorylation of USP20 on Ser334A reduces TRAF6/USP20 association.***A*, mouse SMCs were transduced with recombinant adenoviruses encoding N-terminal HA-tagged USP20 constructs that were WT, S334A, or C154S-H645Q (an inactive enzyme). Twenty-four hours later, SMC protein extracts (25 μg) were incubated with buffer ± 20 pmoles of ubiquitin-VME (Ub-VME) at 37 °C, resolved by SDS-PAGE and immunoblotted with anti-HA IgG, which detected both unmodified USP20 (“native”) and USP20 covalently linked to ubiquitin-VME (“+Ub”). *B*, for each USP20 construct, the densities of bands corresponding to the Ub-VME adduct were normalized to band densities corresponding to the cognate unmodified (“native”) protein (in the samples treated without Ub-VME). These ratios were plotted for three independent experiments (along with means ± SD). Compared with WT and S334A: ∗*p* < 0.05 (one-way ANOVA with Bonferroni post-hoc test). *C*, SMCs were transduced with HA-tagged USP20 isoforms as described in *panel A*. Twenty-four hours later, SMCs were serum starved for 1 h and stimulated with IL-1β (1 ng/ml, 20 min). SMCs were solubilized, and endogenous TRAF6 was immunoprecipitated. Immunoprecipitates (IP) were separated by SDS-PAGE and immunoblotted (IB) serially with antibodies specific for TRAF6 and then HA (for USP20). SMC protein extracts (“lysates”) were serially immunoblotted for HA, phospho-USP20(Ser334) (“p-USP20”), TRAF6, GFP, and β-actin. *D*, the density of USP20 bands in each TRAF6 IP was normalized to the cognate band density for TRAF6; these ratios were obtained for WT and S334A in each experiment and plotted (along with means ± SD) for three independent experiments. Compared with WT USP20: ∗*p* < 0.05. The gel mobility of molecular weight markers (kDa) is indicated beside each *blot panel*. IL-1, interleukin-1; SMC, smooth muscle cell; TRAF, TNFR-associated factor; Ub-VME, ubiquitin vinyl methyl ester; USP20, ubiquitin-specific peptidase 20.

USP20-WT and USP20-S334A both demonstrated equivalent binding to Ub-VME in SMC protein extracts (Fig. 6, *A* and *B*) and thus appeared to have equivalent activity on an idealized substrate. In contrast, Ub-VME did not bind at all to the catalytically inactive USP20 (USP20-C154S/H645Q), in which the active site cysteine and histidine are mutated to serine and glutamine (Fig. 6, *A* and *B*) (3, 29).

USP20 achieves anti-inflammatory activity in SMCs, in part, by deubiquitinating the E3 ubiquitin ligase TRAF6 (3). Could USP20-S334A achieve greater anti-inflammatory activity than USP20-WT *in vivo* (Fig. 4) by deubiquitinating TRAF6 more efficiently? To address this question, we began by assessing the association with TRAF6 with USP20-WT and USP20-S334A in SMCs, because the extent of USP20-mediated deubiquitination depends upon the avidity of USP20/substrate association (3, 11). We immunoprecipitated endogenously expressed TRAF6 from SMCs that had been transduced with equivalent levels of HA-tagged USP20-WT or HA-tagged USP20-S334A and stimulated with IL-1β. TRAF6 immunoprecipitates contained ∼2.2-fold more USP20-S334A than USP20-WT (Fig. 6, *C* and *D*). Thus, it seems that preventing USP20 phosphorylation on Ser334 augments the association of USP20 with its intracellular substrate TRAF6, even though it has no effect on the association of USP20 with the idealized substrate Ub-VME (Fig. 6, *A* and *B*).

**Figure 7:**
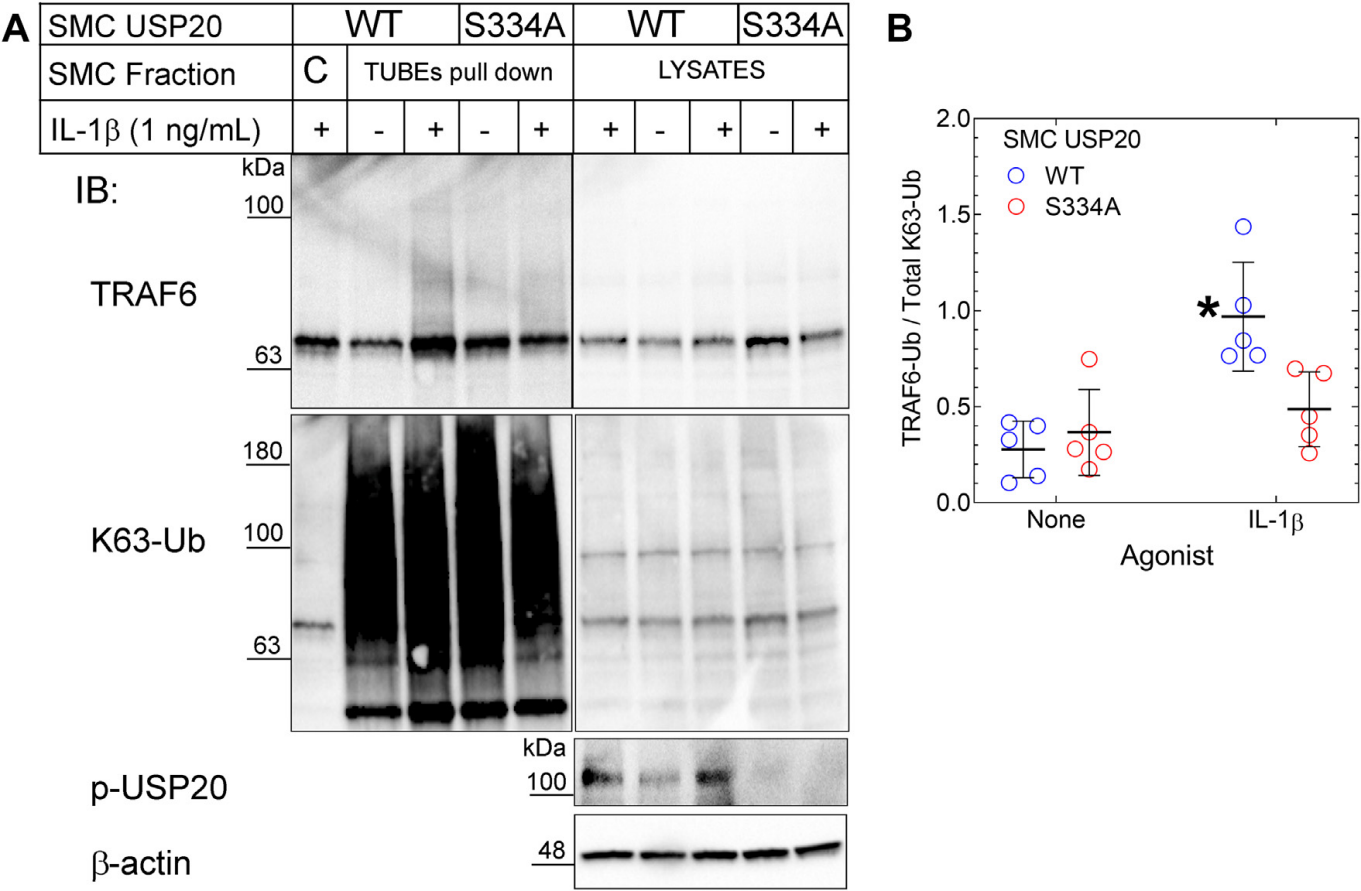
**Phosphorylation of USP20 on Ser334 decreases its activity on TRAF6.***A*, SMCs from WT and USP20-S334A mice were challenged without (“-”) or with (“+”) IL-1β (1 ng/ml) for 20 min and then solubilized. SMC lysates were subjected to polyubiquitin affinity pull-downs using agarose beads lacking (“C”, control) or containing covalently attached TUBEs. Pull-downs and cognate SMC lysates were subjected to serial immunoblotting for TRAF6, K63-linked polyubiquitin (K63-Ub), as well as phospho-USP20(Ser334) (“p-USP20”) and β-actin (lysates only). *B*, for TUBEs pull-downs, ubiquitinated TRAF6 smears (TRAF6-Ub; Mr >60) were normalized to densities of the K63-ubiquitin smears in cognate TUBEs pull-downs; these ratios were plotted along with means ± SD of five experiments with independent SMC lines of each genotype. Compared with unstimulated SMCs: ∗*p* < 0.01 (two-way ANOVA, with Holm-Šídák multiple comparisons test). The gel mobility of molecular weight markers (kDa) is indicated beside each *blot panel*. SMC, smooth muscle cell; TRAF, TNFR-associated factor; TUBEs, tandem ubiquitin binding entities; USP20, ubiquitin-specific peptidase 20.

Because TRAF6 associates more avidly with USP20-S334A than with USP20-WT, one should expect that TRAF6 deubiquitination should be greater in cells expressing USP20-S334A than in cells expressing USP20-WT (3, 11). To address this issue, we used SMCs from USP20-S334A and WT mice. After stimulating these SMCs with IL-1β, we pulled down polyubiquitinated endogenous proteins with tandem ubiquitin binding entities (TUBEs) (4, 30). The TUBEs not only bind polyubiquitin chains with high affinity but also prevent the disassembly of polyubiquitin chains during biochemical isolation (30). We utilized TUBEs comprising four linearly fused ubiquitin-binding domains from ubiquilin; these TUBEs bind K63 polyubiquitin chains with an affinity 8- to 10-fold higher than that with which they bind K48 polyubiquitin chains (30). By immunoblotting SMC TUBEs pull-downs for TRAF6, we found that USP20-WT and USP20-S334A SMCs had equivalent (low) levels of TRAF6 ubiquitination in the absence of inflammatory stimuli. However, WT SMCs demonstrated 2-fold greater TRAF6 ubiquitination than USP20-S334A SMCs in response to IL-1β (Fig. 7). Furthermore, IL-1β-induced ubiquitination of TRAF6 was not even significantly greater than basal levels in USP20-S334A SMCs (Fig. 7). Despite their differences in IL-1β-induced TRAF6 ubiquitination, WT and USP20-S334A SMCs demonstrated equivalent levels of global protein ubiquitination (see lysate blots, Fig. 7). These observations suggest that USP20-S334A does not affect all K63 polyubiquitination mechanisms but rather specifically TRAF6 K63 polyubiquitination. Taken together, these data suggest that both USP20/TRAF6 association and TRAF6 deubiquitination are augmented when USP20 is stabilized in a dephosphorylated state at Ser334.

### USP20-S334A attenuates IL-1β-evoked inflammatory signaling in SMCs

**Figure 8:**
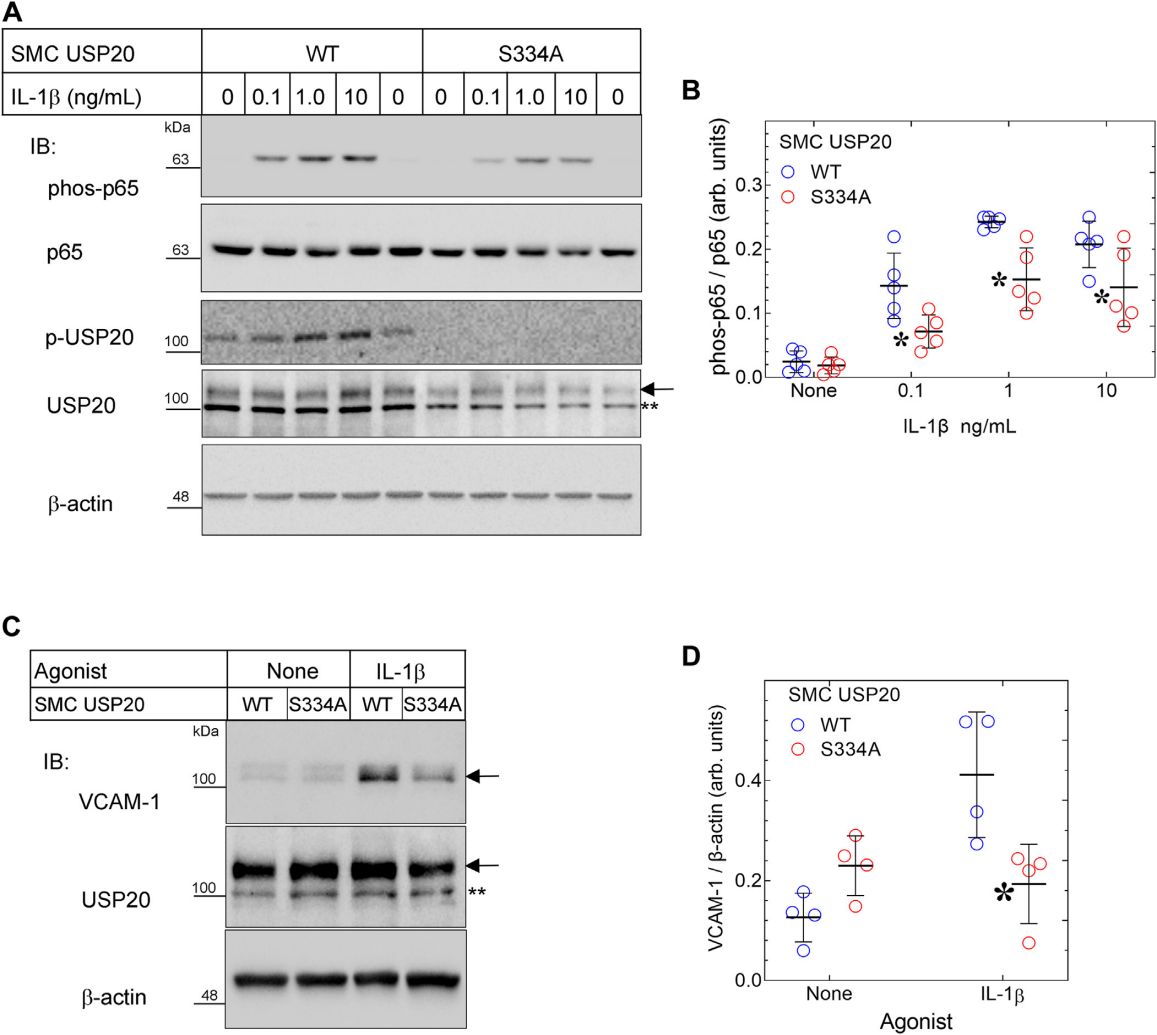
**NFκB signaling induced by IL-1βis diminished in USP20-S334A SMCs.***A*, SMCs from WT and USP20-S334A mice were serum-starved overnight, challenged with the indicated concentration of IL-1β for 20 min (37 °C) and solubilized. Parallel immunoblots of SMC extracts were probed serially with primary IgGs specific for phospho-p65(Ser536) (“phos-p65”), total p65, phospho-USP20(Ser334) (“p-USP20”), total USP20 (designated by the *arrow*; ∗∗ designates a nonspecific band), and β-actin. *B*, the band densities for phos-p65 were normalized to cognate band densities for total p65; these ratios were plotted (along with means ± SD) for five independent experiments with independently isolated WT and USP20-S334A SMC lines. Compared with WT: ∗*p* < 0.05 (two-way ANOVA with Šídák multiple comparisons test). *C*, SMCs from *A* were serum-starved overnight, challenged ± IL-1β (1 ng/ml) for 4 h (37 °C), and solubilized. Immunoblots of SMC extracts were probed serially with IgGs specific for VCAM-1, USP20, and β-actin, as in *panel A*. *D*, the band densities for VCAM-1 were normalized to cognate band densities for β-actin; these ratios were plotted (along with means ± SD) for four independent experiments with independently isolated WT and USP20-S334A SMC lines. Compared with WT: ∗*p* < 0.05 (two-way ANOVA with Šídák multiple comparisons test). The gel mobility of molecular weight markers (kDa) is indicated beside each *blot panel*. IL-1, interleukin-1; SMC, smooth muscle cell; USP20, ubiquitin-specific peptidase 20.

In SMCs, TRAF6 ubiquitination provoked by IL-1β is greatly reduced by expressing physiologic levels of USP20-S334A instead of USP20-WT. Therefore, one might expect that USP20-S334A would also reduce IL-1β-induced NFκB activation, a process that depends upon TRAF6 ubiquitination and activity (1, 3). To test this expectation, we first examined IL-1β-induced phosphorylation of NFκB subunit p65 on Ser536 (21, 22, 23) in SMCs isolated from WT and USP20-S334A mice. In response to IL-1β, p65 phosphorylation on Ser536 was 2-fold greater in WT SMCs than in USP20-S334A SMCs (Fig. 8, *A* and *B*). Concordantly, IL-1β-induced expression of the NFκB-dependent gene product VCAM-1 was 2-fold greater in WT than in USP20-S334A SMCs (Fig. 8, *C* and *D*). Thus, when stabilized in a dephosphorylated conformation, USP20 functions as a highly efficient anti-inflammatory DUB and blunts IL-1β-induced NFκB signaling in SMCs.

### IRAK1 phosphorylates USP20 in purified preparations and in intact SMCs

**Figure 9:**
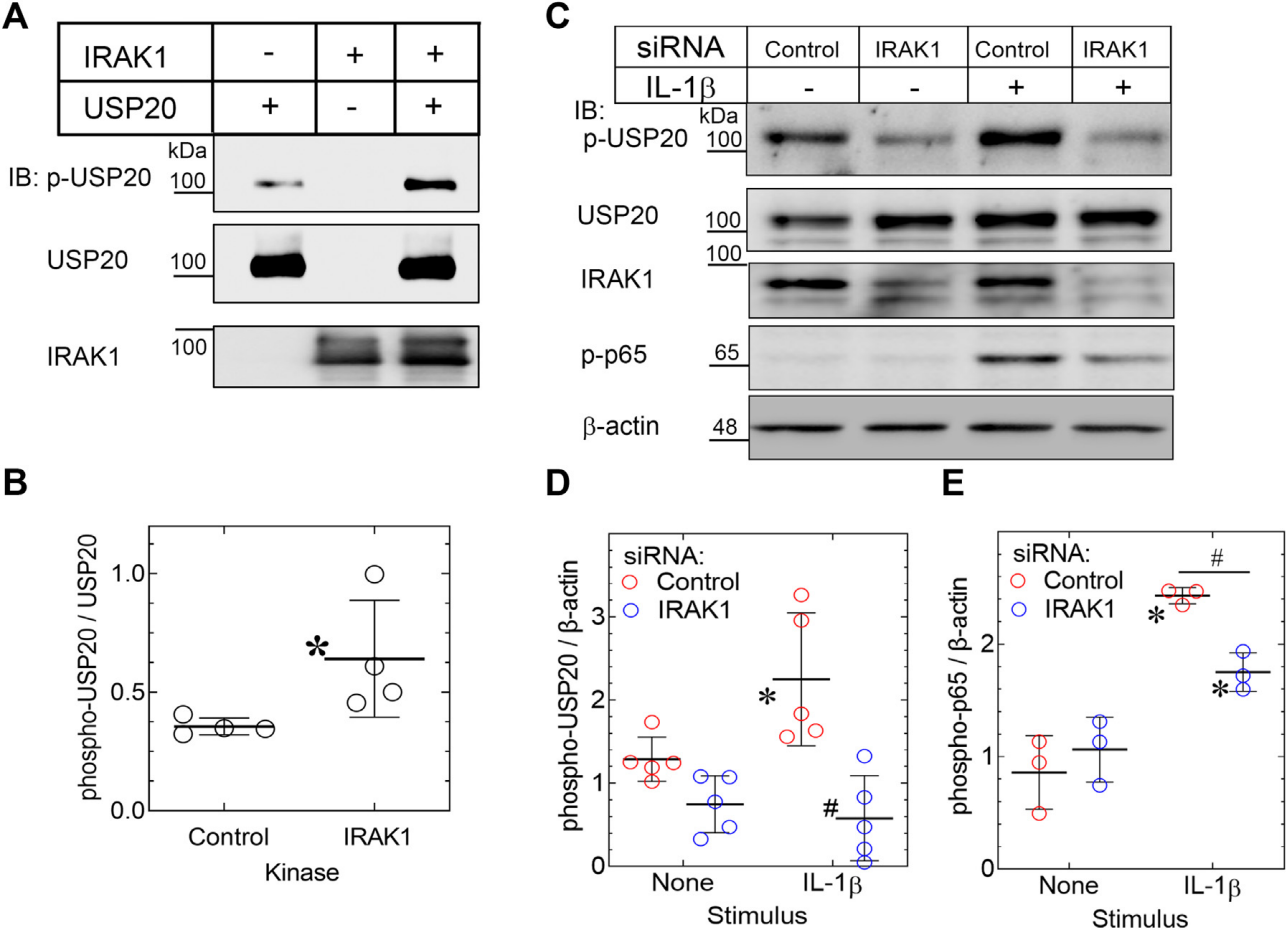
**IRAK1 phosphorylates USP20 on Ser334.***A*, HA-tagged human USP20 was purified from COS-7 cells as described in Experimental procedures, and 0.5 μg was incubated without (“Control”) or with 0.4 μg purified IRAK1 and immunoblotted serially for phospho-USP20 (Ser333) (“p-USP20”), total USP20 and IRAK1. *B*, band densities for p-USP20 were normalized to band densities for cognate total USP20 in the same experiment; ratios were plotted (along with means ± SD from four independent experiments. Compared with control: ∗*p* < 0.01 (unpaired *t* test). *C*, WT SMCs were transfected with siRNA that targets either no mRNA (Control) or IRAK1. Forty-eight hours later, SMCs were serum-starved and treated ± IL-1β (10 ng/ml) for 20 min. Solubilized extracts were immunoblotted (*IB*) serially for phospho-USP20(Ser334) (“p-USP20”), USP20, IRAK1, phospho-p65(Ser536) (“p-p65”), and β-actin. We achieved 70 ± 8% knockdown of IRAK1. *D*, the p-USP20 bands were normalized to cognate β-actin bands; these ratios were plotted as arbitrary units from independent experiments, with means ± SD. Compared with all other samples: ∗*p* < 0.05; compared with IL-1β-stimulated control SMCs: #*p* < 0.001 (2-way ANOVA with Sidak’s post-hoc test). *E*, the p-p65 bands were normalized to cognate β-actin bands and plotted as in *panel D*. Compared with unstimulated control samples: ∗*p* < 0.05; Compared with control siRNA-transfected SMCs challenged with IL-1β: #*p* < 0.05 CTL (2-way ANOVA with Sidak’s post-hoc test). The gel mobility of molecular weight markers (kDa) is indicated beside each *blot panel*. IL-1, interleukin-1; SMC, smooth muscle cell; USP20, ubiquitin-specific peptidase 20.

Residing within the motif RMKDRKFS^334^WGQQRTN, the Ser334 of USP20 is phosphorylated by PKAα—even though the amino acid sequence slightly diverges from the canonical RRxS PKA motif (10, 11). We hypothesized that in the context of IL-1β signaling, perhaps other serine/threonine kinases, including IRAK1 might also phosphorylate USP20 Ser334. The serine/threonine kinase IRAK1 is a key mediator of TRAF6 activation downstream of TLR4 and the IL-1R. Therefore, IRAK1 could not only promote TRAF6 activation but also prevent TRAF6 inactivation (deubiquitination)—if, indeed, IRAK1 phosphorylates USP20 on Ser334 (and thereby reduces the association of USP20 with TRAF6). To test this possibility, we used purified IRAK1 to phosphorylate human USP20 purified from COS-7 cells (29). Phosphorylation of human USP20 on Ser333 in the absence of purified kinase was attributed to the activity of one or more kinases in the COS-7 cells. However, incubation of this purified USP20 with IRAK1 increased human USP20 Ser333 phosphorylation >2-fold, as detected by our phospho-USP20(Ser333) IgG (Fig. 9, *A* and *B*). Thus, IRAK1 can phosphorylate USP20 on Ser333 (of the human USP20 sequence) in preparations of purified proteins.

Does IRAK1 phosphorylate mouse USP20 on Ser334 in intact SMCs? To address this question, we used RNAi targeting IRAK1 in WT mouse SMCs. Phosphorylation of USP20 on Ser334 increased 2.5-fold in response to IL-1β in SMCs transfected with control siRNA (Fig. 9, *C* and *D*). In contrast, IL-1β-induced phosphorylation of USP20 on Ser334 was abolished in SMCs transfected with IRAK1-targeting siRNA (Fig. 9, *C* and *D*). Furthermore, by silencing IRAK1, we also reduced basal levels of phospho-USP20(Ser334) (Fig. 9, *C* and *D*). As shown in Fig. S5, IRAK1 silencing did not lead to a reduction in the expression levels of PKAα and PKAβ isoforms in SMCs. Hence, we infer that IRAK1 knockdown is specific in SMCs, and SMC IRAK1 phosphorylates USP20 Ser334. As one would expect on the basis of IRAK1’s importance to IL-1R-triggered signaling, IRAK1-silenced SMCs demonstrated significantly less IL-1β-evoked NFκB activation, assessed as phosphorylation of p65 on Ser536 (Fig. 9, *C* and *E*). Furthermore, we have seen in Figures 7 and 8 that preventing phosphorylation of USP20 on Ser334 reduces TRAF6 ubiquitination and NFκB activity. Together, these results suggest that IRAK1 inhibits USP20-mediated deubiquitination of TRAF6 by phosphorylating USP20 on Ser334 and reducing USP20:TRAF6 association. It therefore seems likely that IL1-β-induced inflammatory signaling can be suppressed by preventing USP20 phosphorylation on Ser334.

## Discussion

**Figure 10:**
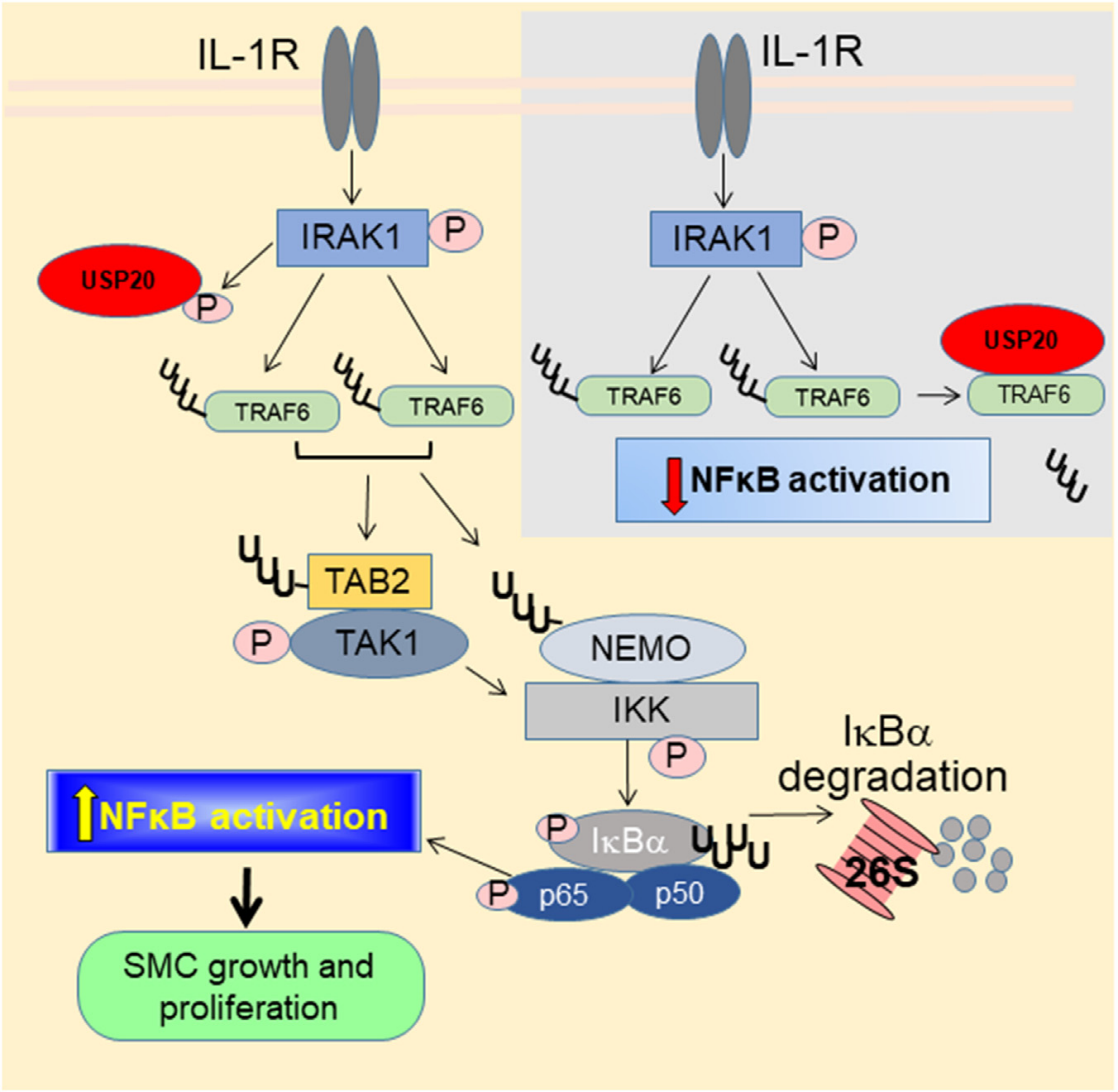
**Effects of USP20 Ser334 phosphorylation in IL-1R signaling.** Activation with IL-1β promotes dimerization of the IL-1R and subsequent recruitment and activation of the IL-1R-associated kinase1 (IRAK1), which is a key mediator of IL-1R signaling. Activated IRAK1 binds to and activates the adaptor called tumor necrosis factor associated factor 6 (TRAF6). TRAF6 autoubiquitinates as part of its activation process, and subsequently ubiquitinates NFκB essential modulator (NEMO) as well as the TAB2 subunit of the TAK1 kinase complex—both by covalently attaching K63-linked polyubiquitin and by synthesizing free K63-linked polyubiquitins that serve as substrates for generating hybrid K63/linear ubiquitin chains that bind to NEMO and TAB2 *via* their ubiquitin-binding domains and subsequently augment their activity (not shown). TAK1 then autophosphorylates, activates and then phosphorylates and activates IκB kinase-β (IKK). Phosphorylation of IκBα by IKK provokes K48-linked polyubiquitination and 26S proteasomal degradation of IκBα. Subsequently, the now-uninhibited protein subunits of NFκB translocate to the nucleus and trigger gene transcription that promotes SMC proliferation and migration. In this signaling cascade, IKK also phosphorylates the p65 subunit of NFκB (and thereby increases its transcriptional activity (4)). Unphosphorylated USP20 (*gray inset*) deubiquitinates and thereby deactivates TRAF6 and thus diminishes downstream activation of NFκB. When IRAK1 phosphorylates USP20 Ser334 (schema at *left*, *yellow-shaded* background), the association of USP20 with TRAF6 is weakened and therefore USP20-mediated deubiquitination and deactivation of TRAF6 is prevented; signaling to NFκB activation proceeds. Preventing USP20 Ser334 phosphorylation (as in USP20-S334A) augments the binding of USP20 to TRAF6, augments TRAF6 deubiquitination, and diminishes NFκB activation (either by deubiquitinating and thereby inactivating a subset of TRAF6 proteins or by reducing the ubiquitination level of most or all TRAF6 proteins). UUU denotes K63-linked polyubiquitin; ^U^_U_^U^_U_ denotes K48-linked polyubiquitin. IL-1, interleukin-1; SMC, smooth muscle cell; USP20, ubiquitin-specific peptidase 20.

This work demonstrates that blocking phosphorylation of USP20 on Ser334 augments the anti-inflammatory activity of USP20 on IL-1β signaling (Fig. 10). In preparations of purified proteins and in intact SMCs, we found that phosphorylation of USP20 on Ser334 is mediated by IRAK1. Thus, IRAK1 not only transduces IL-1R signaling (1) but also prevents its termination: phosphorylation of USP20 on Ser334 reduces USP20’s binding to TRAF6 and thereby diminishes (a) USP20-mediated deubiquitination/deactivation of TRAF6 and (b) consequent attenuation of downstream NFκB activation.

USP20 is a member of the USP subfamily of DUBs, which are endowed with protein interaction domains that also participate in the activation process of these enzymes (31). Consequently, DUBs are expressed not in a precursor form but rather as fully processed enzymes; substrate recognition and substrate binding play major roles in triggering DUB activity (31). In addition to this substrate-dependent activation, other modes of DUB regulation exist: incorporation into large macromolecular complexes that facilitate DUB activity; inactivation by autoproteolysis; and site-specific dynamic posttranslational modification of the DUB itself by ubiquitination, phosphorylation, and *0*-glycosylation (32, 33, 34, 35).

The phosphorylation/dephosphorylation of USP20 on Ser334 appears to regulate USP20 protein–protein interactions without affecting protein stability or subcellular localization (10, 11). When Ser334 remains dephosphorylated, USP20’s association with TRAF6 (this study) and the β_2_-adrenergic receptor (10) is favored, whereas when Ser334 is phosphorylated, association with the β_1_-adrenergic receptor is favored (11). It remains to be determined whether phosphorylation of Ser334 regulates the association of USP20 with other known USP20 substrates, including type 2 iodothyronine deiodinases, hypoxia-inducible factor-1α, Rad17, claspin, pyruvate kinase isozyme M2, extracellular signal regulated kinase 3, ULK1, and β-catenin (36, 37, 38, 39, 40, 41, 42, 43). The Ser334 phosphorylation status of USP20 affects neither the binding of USP20 to the active-site directed probe Ub-VME nor USP20-mediated deubiquitination *per se*: whereas phosphorylation of USP20 on Ser334 reduces USP20-mediated deubiquitination of the β_2_-adrenergic receptor, it increases USP20-mediated deubiquitination of the β_1_-adrenergic receptor (10, 11).

The exact mechanisms that define substrate-specific activity of USP20 remain unknown and would require future in-depth studies and/or structures of USP20-protein complexes. Ser334 phosphorylation by either PKA (10, 11) or IRAK1 (Fig. 9) does not impact USP20’s protein stability. In contrast, phosphorylation of USP20 at sites distinct from Ser334 does affect USP20 stability. The DNA-damage response induced by prolonged UV exposure or hydroxyurea treatment activates ataxia-telangiectasia mutated (ATM) and ATM and Rad3 related (ATR) kinases, leading to phosphorylation of USP20 at four potential SQ/TQ motifs in hUSP20: T170Q, T232Q, S305Q and S662Q (43). When USP20 is thus phosphorylated, the association of USP20 with the E3 ubiquitin ligase HERC2 diminishes and, consequently, K48 polyubiquitination of USP20 diminishes. As a result, proteasomal degradation of USP20 diminishes and its half-life increases (43, 44). ATM/ATR-mediated phosphorylation of USP20 does not affect its DUB activity; rather, this USP20 phosphorylation enhances the association of USP20 with Rad17 and thereby enhances USP20-mediated deubiquitination of Rad17 (41).

Phosphorylation of USP20 appears to activate USP20-mediated deubiquitination of HMG-CoA reductase (HMGCR) in the hepatocyte (45). When it is phosphorylated on Ser132/Ser134 by mTORC1, USP20 associates with the ubiquitin E3 ligase autocrine motility factor receptor (GP78). Autocrine motility factor receptor itself associates with HMGCR and scaffolds USP20 with its substrate HMGCR (45).

Our studies revealed that USP20 Ser334 is phosphorylated by IRAK1. Nonetheless, the USP20 motif containing Ser334 does not conform to the Pro-S/T motif typical of IRAK1-phosphorylated sites in several other known IRAK1 substrates (46, 47). Scrutiny of USP20 protein sequences revealed the presence of additional Ser/Thr residues flanked by prolines, which may be phosphorylated by IRAK family kinases. It remains to be determined whether these sites affect the role of IRAK1 in modulating USP20-mediated deubiquitination in NFκB signaling.

*In vivo*, neointimal hyperplasia was reduced by ∼55% in mice that expressed USP20-S334A instead of USP20-WT. This result contrasts with that obtained when we overexpressed USP20-WT in SMCs (3): SMC-specific overexpression of USP20-WT had no effect on neointimal hyperplasia. However, SMC-specific overexpression of dominant-negative USP20 augmented neointimal hyperplasia 2.6-fold (3). These apparently paradoxical results may be reconciled by considering the regulatory role played by phosphorylating USP20 on Ser334. Phosphorylation of USP20 on Ser334 substantially attenuates the anti-inflammatory activity of USP20-WT, whether USP20-WT expression is physiologic or supraphysiologic. Antagonizing USP20 with a dominant-negative mutant vitiates the anti-inflammatory activity of USP20, but preventing phosphorylation of USP20 on Ser334 augments the anti-inflammatory activity of USP20.

Genetic and pharmacologic interventions that mitigate neointimal hyperplasia almost always mitigate atherosclerosis—as we (3, 4, 12, 16, 17, 48, 49, 50) and others (51, 52, 53, 54, 55, 56, 57, 58, 59, 60, 61, 62, 63) have shown, including with USP20 itself (3, 4). Whether targeting USP20-Ser334 phosphorylation therapeutically could mitigate inflammatory vascular diseases like atherosclerosis remains an intriguing possibility.

## Experimental procedures

### Materials

N-ethylmaleimide was obtained from Sigma-Aldrich. Murine IL-1β was purchased from PeproTech, Inc. Antibodies used for immunoblotting and immunofluorescence are listed below, along with their sources. All antibodies were diluted at 1:1000 for immunoblotting and 1:50 for immunofluorescence, unless stated otherwise. In parentheses, we list the catalog number and the dilution used for immunoblotting/dilution used for immunofluorescence: mouse monoclonal anti-β-actin (#A5441, 1:10,000), Cy3-conjugated mouse IgG specific for ACTA2 (clone 1A4; #C6198, 1:1000) or V5 (#V4014, 1:1000), mouse IgG specific for collagen I (#C2456, 1:100), from Sigma-Aldrich; rabbit polyclonal anti-USP20 (#A301-189A, 1:3000) from Bethyl Laboratories, Inc; rabbit polyclonal anti-phospho Ser333 USP20 (custom generated, GenScript, 1:3000 for Western blots, 1:50 for immunofluorescence) (10); rabbit IgG specific for von Willebrand factor (#A0082, 1:100) from Agilent Dako; rabbit monoclonal anti-phospho-p65(Ser536) (#3033, 1:1000 for Western blots, 1:100 for immunofluorescence), rabbit monoclonal anti-cleaved caspase-3 (#9664, 1:100), rabbit monoclonal anti-HA (#C29F4, 1:1000), rabbit monoclonal anti-TRAF6 (E2K9D, 1:1000) from Cell Signaling Technologies; anti-IRAK1 (sc-7883, 1:500), anti-NFκB p65 (#sc-372, 1:500 for Western blots, 1:100 for immunofluorescence), anti-VCAM-1 (#sc-8304, 1:1000 for Western blots, 1:100 for immunofluorescence), and anti-PCNA (#sc-7907) from Santa Cruz Biotechnology; anti-green fluorescent protein (MBL-598, 1:3000) from MBL LifeSciences; rabbit polyclonal anti-K63-linked polyubiquitin (clone APU3, #05-1308, 1:1000), from Millipore.

Horseradish peroxidase-conjugated secondary antibodies were from Cell Signaling Technology, Cytiva, and Bethyl Laboratories, Inc; they were used at a dilution of 1:3000. Secondary antibodies conjugated to Alexa Fluor 488 and Alexa Fluor 546 were obtained from Invitrogen; they were used at 1:100.

Agarose-TUBEs (UM401) and uncoupled agarose (UM400) were purchased from LifeSensors, Inc.

### Mice

All animal experiments were performed in accordance with protocols approved by Duke University Institutional Animal Care and Use Committee. All mice were congenic on the C57BL/6J genetic background. We used gene editing with CRISPR/Cas9 to generate C57BL/6J mice with USP20-S334A (to mimic unphosphorylated USP20) (64, 65, 66, 67, 68). To insert the desired mutation in USP20, single guide RNA GCTCTGAGTTGGTGCGCTGCTGG was generated by *in vitro* transcription of a short DNA template following a published protocol (69). The following 200-nt single stranded oligodeoxynucleotide repair oligo was used for mutating Ser334 to alanine: cgtggtggaggcagctcaaaggccgagatggagctgctgatctcagatgaggcgggccgagccatctctgagaaggagcggatgaaggaccgcaagttcGcctggggTcagcagcgcaccaactcagagcaagtggatgaggatgcagatgtggacacggccatggcgtcccttgatgagcaatccagagaggcccagcc. Cas9 protein (Alt-R Cas9 v3 Cat#1081059, IDT) was used to form single guide RNA/Cas9 Ribonucleoprotein complex, which along with the repair oligo was electroporated into C57/BL6J mouse embryos following a published protocol (69). Oviduct implantation of embryos to obtain litters with potential founders was carried out as described (69). Founders were screened by genomic PCR, subcloning, and sequencing analyses. The following primers were used for PCR and sequencing to confirm desired mutation (Fig. S1). Forward primer: 5′ggagttcctacgctgcctaatgg3′; reverse primer: 5′cctcaccctctagcatgttagtg3′. We obtained germline transmission of USP20-S334A (16 positive out of 40 F1 pups), and the USP20-S334A mice were healthy and viable. We cross-bred USP20-S334A heterozygous mice to obtain homozygous pups and WT littermate controls that were used in our experiments. All mice were verified by genotyping using PCR and sequencing steps as shown in Fig. S1.

### Mouse physiology

Mouse BP and heart rates were obtained on anesthetized and instrumented mice with the help of Duke Cardiovascular Research Center physiology core facility using previously reported methods (70, 71).

### Histology

With approval from the Duke University School of Medicine Institutional Review Board, human peroneal arteries were obtained as discarded surgical materials from de-identified leg amputations at Duke Hospital, as described (12). The arterial samples from each patient were divided into segments that were judged by tactile and visual inspection to be (a) atherosclerotic but not calcified (firm, with luminal narrowing), and (b) minimally atherosclerotic or nonatherosclerotic (supple, with minimal or no apparent luminal narrowing). Atherosclerotic and nonatherosclerotic arterial segments from each patient were embedded in paraffin, sliced at 5 μm, and immunostained pairwise (see below) (12).

Mouse carotid arteries were embedded in Neg-50 (Thermo Fisher) and frozen (12). Frozen specimens were sliced at 5 μm and stained with hematoxylin and eosin (16, 17). Computerized planimetry with Image J was performed by observers blinded to sample identity to calculate the cross-sectional area for the neointima, tunica media, and lumen, as described (12).

Immunofluorescence was performed on 5-μm tissue frozen sections (50). Tissue sections were fixed for 5 min (RT) in 15% formalin/75% ethanol (v/v), then washed twice in PBS (5 min/wash) and three times in “TBS-T”: 15 mM Tris-Cl pH 8.0 (RT), 150 mM NaCl, and 0.2% (v/v) Tween-20. Sections were blocked (RT, 30–60 min) with PBS containing 5% bovine serum and 0.2% (w/v) gelatin. After three washes with TBS-T (2 min/wash), specimens were incubated with the primary IgG diluted in 0.2% (w/v) gelatin in PBS for 1 h at RT. After two washes with TBS-T (5 min/wash), tissue sections were incubated with secondary IgG (and Hoechst 33342—see below) in 0.2% gelatin/PBS for 45 to 60 min at RT. After two washes of 5 min in TBS-T, tissue slices were covered with Fluoromount-G (SouthernBiotech), coverslipped, and imaged.

Tissue sections were probed with rabbit IgGs specified above, followed by anti-rabbit IgG conjugated to Alexa-488 or Alexa-548, as described (50). Secondary IgG incubations included Hoechst 33342 (10 μg/ml) to counter-stain nuclear DNA. In select cases, secondary IgG incubations included Cy3-conjugated anti-ACTA2 (or isotype control) IgG, as described (50). Images were obtained with a Leica TCS SP8 confocal microscope adjusted to an optical slice thickness of 1 μm (630 × original magnification); identical camera settings were used for all samples within each staining cohort (including negative controls). Colocalization of green (PCNA) with blue (DNA) was performed using Imaris 9.2 software and pseudocolored white. Nonspecific fluorescence (determined on serial sections probed with equivalent concentrations of nonimmune rabbit IgG) was subtracted from total signal to obtain antigen-specific fluorescence. Imaging and analyses were performed by observers blinded to specimen identity. Although single high-magnification images are shown in the figures, quantitation of protein fluorescence was performed on complete carotid artery cross-sections, which were compiled photographically from 4 to 6 200 × (original magnification) images per specimen; these four to six images per specimen were digitally stitched together into a single image per specimen with Imaris 9.2 software. For each staining experiment, WT and USP20-S334A samples were stained and imaged batch-wise.

### Carotid endothelial denudation

Mice were anesthetized with pentobarbital (50 mg/kg, i.p.). Subsequently, endothelial denudation was performed on the common carotid artery *via* the external carotid artery with a 0.36-mm-diameter coronary guidewire (Cordis), as we described (12). Four weeks after endothelial denudation, injured common carotids were harvested from anesthetized mice after 20 min of perfusion-fixation (80 mm Hg) with 10% formalin in PBS; subsequently, carotids were incubated in 30% (w/v) sucrose in PBS, embedded in Neg-50, and frozen at −80 °C.

### Cell lines

Aortic SMCs were isolated from the descending thoracic aortas of WT and USP20-S334A mice matched for age and sex. For a single line of primary SMCs, we typically pooled aortas from 2 to 3 mice per genotype. The aortas were stripped of adventitia and endothelial cells and then subjected to digestion with collagenase and elastase, as described (12). SMCs were split 1:4 for each passage, and not used after passage 6 (freshly isolated cells = passage 1). All SMC experiments were performed with ≥3 independently isolated lines of WT and USP20-S334A SMCs (12). SMC growth medium comprised 10% (v/v) fetal bovine serum and 1% (v/v) penicillin/streptomycin (Gibco) in Dulbecco’s modified Eagle medium (DMEM). Serum-free medium comprised DMEM with 20 mM Hepes, pH 7.4 and 0.1% (w/v) fatty acid-free bovine serum albumin (Sigma-Aldrich).

Human embryonic kidney (HEK-293) and COS-7 cells were obtained from the American Type Culture Collection. These cells were grown in minimal essential medium (HEK-293) and DMEM (COS-7) supplemented with 10% fetal bovine serum and 1% penicillin/streptomycin.

### SMC proliferation and migration

SMC proliferation in 96-well plates was quantitated with a dye-binding assay as described (12). SMC migration through Transwell membranes (8-μm pore) was assayed as described (12).

### RNA interference

Small-interfering RNA targeting mouse IRAK1 (Dharmacon GE Healthcare Life Sciences) or no known mouse RNA (control) were synthesized with the following sequences (sense strand is listed): 5′-AAUUCUCCGAACGUGUCACGU-3′ (control); 5′-CGAGCAGUCAUGAGAAAUAUU-3′ (IRAK1). SMCs were transfected with 20 μg per 100 mm dish or 3.3 μg per well of 6-well dish of either control or IRAK1-targeted siRNA in serum-free medium using Lipofectamine 2000 (Thermo Fisher Scientific) according to the manufacturer’s protocol. After 24 h at 37 °C, SMCs received serum-containing medium, which was replaced 24 h later with serum-free medium. After overnight incubation in serum-free medium, SMCs were stimulated with IL-1β for the indicated times.

### Adenovirus-mediated transduction

Recombinant adenoviruses expressing HA-tagged USP20 were generated in our laboratory using the AdEasy system (Agilent Technologies). Control eGFP virus was purchased from Vector Biolabs. The HA-tagged mouse USP20 cDNA (3) was first cloned into the pShuttle-CMV vector, after which it was mobilized into the pAdEasy-1 vector by homologous recombination according to established protocols (72). All plasmid constructs were verified by sequencing to ensure the integrity of the HA-USP20 cDNA. Linearized pAdEasy-HA-USP20 plasmid was transfected into HEK-293 cells and the released HA-USP20 adenoviral particles were collected and serially amplified in HEK-293 cells. High-titer adenovirus was purified from HEK-293 cell lysates and supernatants by PEG precipitation followed by cesium chloride purification (72). Adenoviral transductions of SMCs were performed as reported previously (4).

### Immunoprecipitation and immunoblotting

SMCs were solubilized in an ice-cold “lysis buffer”: 50 mM Hepes (pH 7.5), 2 mM EDTA, 250 mM NaCl, 10% (v/v) glycerol, 0.5% (v/v) Nonidet P-40, supplemented with 10 mM N-ethylmaleimide, as well as with phosphatase and protease inhibitors (1 mM sodium orthovanadate, 10 mM NaF, 100 μM phenylmethylsulfonyl fluoride, 5 μg/ml leupeptin, 5 μg/ml aprotinin, 1 μg/ml pepstatin A, and 1 mM benzamidine). Co-immunoprecipitation assays were performed overnight using anti-TRAF6(D-10)–conjugated agarose (Santa Cruz Biotechnology, # SC-8409 AC). After overnight end-over-end rotation at 4 °C, the protein complexes were washed three times with lysis buffer, eluted in 2 × SDS-PAGE protein sample buffer, and resolved on 4 to 20% gradient Tris-glycine gels along with 20 μg of the corresponding lysates (in parallel lanes), which represented approximately 2% of the cell protein subjected to IP. Proteins were transferred onto nitrocellulose membranes, which were blocked with 5% (w/v) dried skim milk powder in TTBS buffer [0.2% (v/v) Tween 20, 10 mM Tris-Cl (pH 8.0 at 25 °C), 150 mM NaCl]. Incubations of nitrocellulose membranes with IgG (45 min, 25 °C) were performed in the same buffer; nitrocellulose washes were performed in protein-free TTBS. Proteins were detected with enhanced chemiluminescence (SuperSignal West Pico Reagent, Pierce) using a charge-coupled device camera system (Bio-Rad Chemidoc-XRS). Densitometry of protein bands was performed with Image Lab software (Bio-Rad).

### Polyubiquitinated protein pull-down

SMCs were solubilized in lysis buffer, and high-affinity pulldown of polyubiquitinated proteins was performed using agarose beads to which either no protein or TUBEs (1, UM401) were covalently linked, according to the manufacturer’s protocol. Cell lysates were processed as mentioned in the co-immunoprecipitation experiments above, except that washing TUBEs–agarose affinity complexes was performed with TTBS before elution.

### USP20 purification

USP20 was purified by immunoaffinity chromatography, as we reported (73). Briefly, COS-7 cells were transfected with either pcDNA3-HA or pcDNA3-HA-USP20. Cells were lysed in glycerol NP40 lysis buffer lacking EDTA and N-ethylmaleimide. Insoluble debris was pelleted, and supernatants were incubated and mixed overnight at 4 °C with anti-HA IgG-agarose beads (Pierce Anti-HA Agarose, Thermo Fisher Scientific, #26182). Immune complexes were washed three times; resin-bound HA-USP20 was eluted from beads after incubation (1 h, 4 °C) with HA peptide: 60 to 100 μg/ml in 50 mM Tris-Cl pH 8.0 (25 °C), supplemented with 0.5 × protease inhibitors (see above). Eluted protein was concentrated by membrane ultrafiltration with Vivaspin spin columns (50 kDa cutoff; Vivascience). About 100 to 250 μg of HA-USP20 enzyme were obtained from monolayer COS-7 cells collected from six 150-mm dishes; purity was >80% by SDS-PAGE (4).

### Quantitative RT-PCR

Total RNA was isolated from SMCs using TRIzol (Thermo Fisher) according to the manufacturer’s protocol. Complementary DNA was prepared from the total RNA using the iScript cDNA synthesis kit (Bio-Rad). Primers for determining RNA expression levels of *Tlr4*, *Il1r*, *Tnfr*, *Tak1*, *Myd88*, *Asc*, and the housekeeping gene *Gapdh* were as described (74, 75, 76). Primers used are listed in Table S1. Real-time quantitative RT-PCR was performed using Power SYBR Green PCR Master Mix (Thermo Fisher) and was carried out using the Applied Biosystems StepOnePlus Real-Time PCR System (76). Threshold count values were normalized to GAPDH to calculate fold changes in mRNA expression compared to mean control values by the ^ΔΔ^Ct method (77). Samples were measured in quadruplicate within each assay.

### Statistical analyses

All experiments were performed at least three independent times. Data from three or more independent experiments were averaged and presented as means ± SD. Statistical comparisons between two groups were made with 2-tailed *t* tests as noted in Figure legends. Comparisons among more than two groups and analyses of time course data were made with two-way ANOVA followed by the Sidak post-hoc test for multiple comparisons, unless otherwise noted in the legend, using GraphPad Prism 9.2 software (GraphPad, Inc). Statistical significance was set at *p* < 0.05.

## Data availability

All data are included in the manuscript or are available from the corresponding authors Drs Freedman and Shenoy.

## Supporting information

This article contains supporting information.

## Conflict of interest

The authors declare that they have no conflict of interest with the contents of this article.
