# Supplementary material for: Phosphorylation of USP20 on Ser334 by IRAK1 promotes IL-1β-evoked signaling in vascular smooth muscle cells and vascular inflammation
Source: J Biol Chem. 2023 Jun 11;299(7):104911. doi: 10.1016/j.jbc.2023.104911 (PMC10362797; doi:10.1016/j.jbc.2023.104911)
Supplement: Supporting Table S1 and Figures S1–S5 [file mmc1.pdf]

Phosphorylation of USP20 on Ser334 by IRAK-1 Promotes IL-1β-evoked Signaling in Vascular Smooth Muscle Cells and Vascular Inflammation

Lisheng Zhang[‡], Jiao-Hui Wu[‡], Pierre-Yves Jean-Charles[‡], Pavitra Murali[‡], Wenli Zhang[‡], Aeva Jazic[‡], Suneet Kaur[‡], Igor Nepliouev[‡], Jonathan A. Stiber[‡], Kamie Snow[‡], Neil J. Freedman[‡†1] and Sudha K. Shenoy[‡†2]

From the Departments of [‡]Medicine (Cardiology) and [†]Cell Biology,
Duke University Medical Center, Durham, North Carolina 27710
[1]To whom correspondence may be addressed: Box 102150, Duke University Medical Center, Durham, NC 27710. Tel.: 919-684-6873; Fax: 919-681-0718; E-mail: neil.freedman@duke.edu
[2]To whom correspondence may be addressed: Box 103204, Duke University Medical Center, Durham, NC 27710. Tel.: 919-681-5061; Fax: 919-681-7851; E-mail: skshenoy@dm.duke.edu

SUPPLEMENTARY INFORMATION

TABLE S1. Primers used for qRT-PCR

Gene	Forward Primer (5' to 3')	Reverse Primer (5' to 3')
Tlr4	TGCATGGATCAGAAACTCAGC	ATTGTTTCAATTTCACACCTGGA
Il1r	TCATATGCAGGCTTTAAGGAGAA	GGTGTCGCCGTGCATTTTAT
Tnfr1	CTAAGTGTCCTCCTGGCCAAT	TGGGTTTTCAAGGCGCAGTA
Myd88	TGTTCTTGAACCCTCGGACG	TTCTGGCAGTCCTCCTCGAT
Tak1	TCTGCCAGTGAGATGATCGAA	TCCATCACAAGACATACTGGATTC
Asc	TGACAGTGCAACTGCGAGAA	TGGTCCACAAAGTGTCCTGT

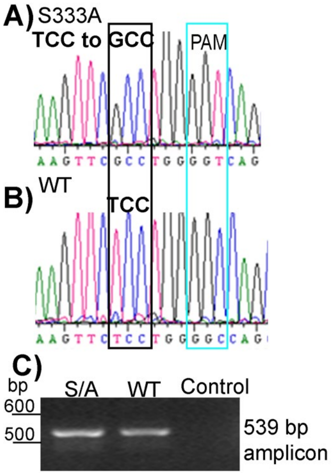

Figure S1. Creation and characterization of the USP20-S334A mouse. *A*, CRISPR/Cas9 technology was employed to edit Ser334 (TCC) to Ala334 (GCC). Shown is a sequence chromatogram of the TCC-to-GCC mutation, confirmed by founder analysis and allelic subcloning. *B*, The WT sequence TCC is shown. Panel *A* also shows the engineered silent mutation (GGC to GGT) included in the repair oligo, designed for the purpose of disrupting the Protospacer Adjacent Motif (PAM) sequence. *C*. PCR amplification of no DNA ("control") or DNA from USP20-S334A ("S/A") and WT mice was followed by agarose electrophoresis. bp: base pairs.

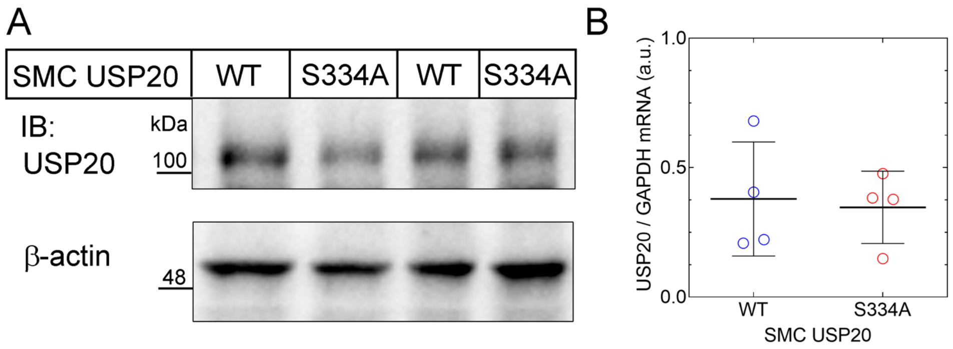

Figure S2. Expression of USP20 protein is equivalent in SMCs from WT and USP20-S334A mice. *A,* Primary SMCs were prepared from WT or USP20-S334A mice, and protein extracts (40 µg) were separated on 4-12 % gradient gels and serially immunoblotted for USP20 and β-actin. *B,* The intensity of USP20 bands was normalized to cognate β-actin bands and plotted along with means ± SD from 4 independently isolated SMC lines of each genotype.

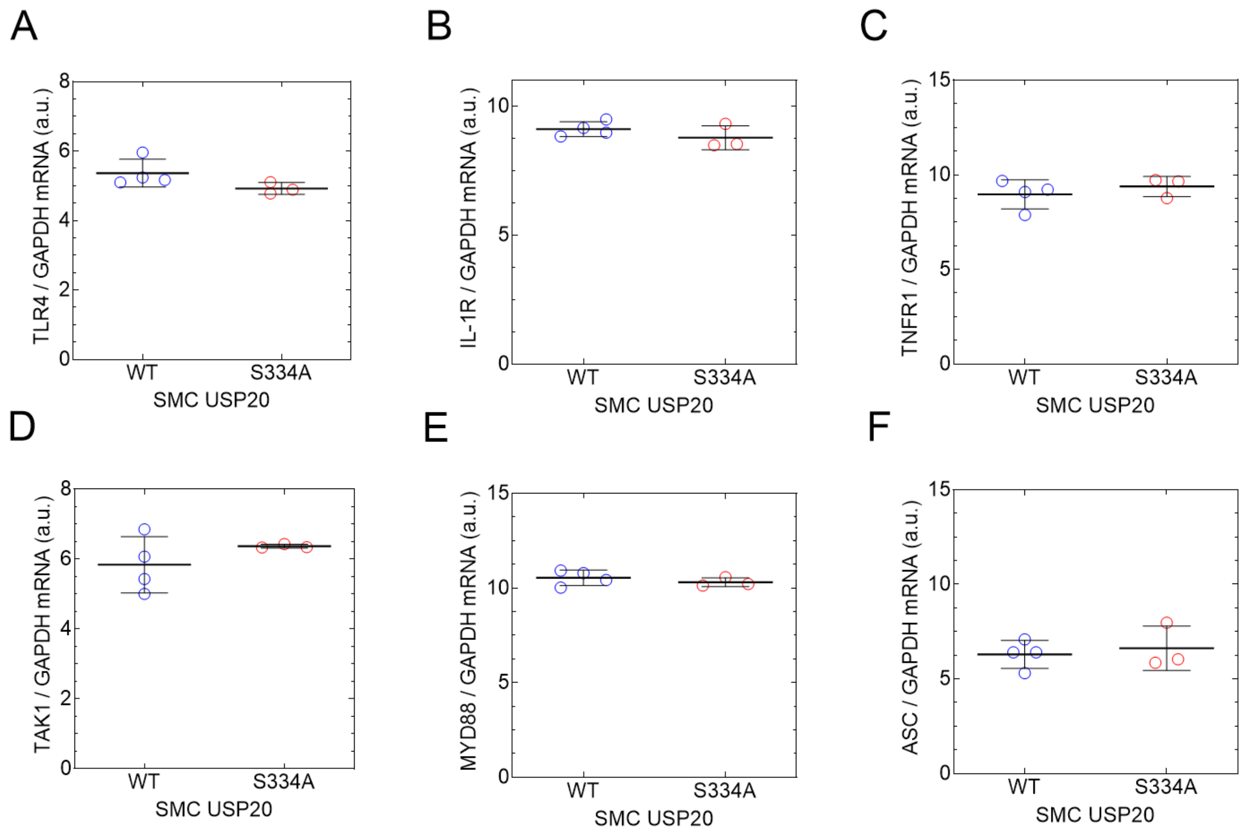

Figure S3. SMCs from WT and USP20-S334A mice express equivalent levels of pro-inflammatory signaling intermediates. Total mRNA was isolated from 4 WT and 3 S334A SMC cell lines and analyzed by qRT-PCR for the expression of TLR4 (A), interleukin-1 receptor (IL1-R) (B), TNF receptor-1 (TNFR1) (C), TAK1 (D), MyD88 (E), and ASC (F) using gene-specific primers (see methods). The level of each mRNA was normalized to cognate levels of GAPDH mRNA, and ratios were plotted (with means ± SD) as arbitrary units.

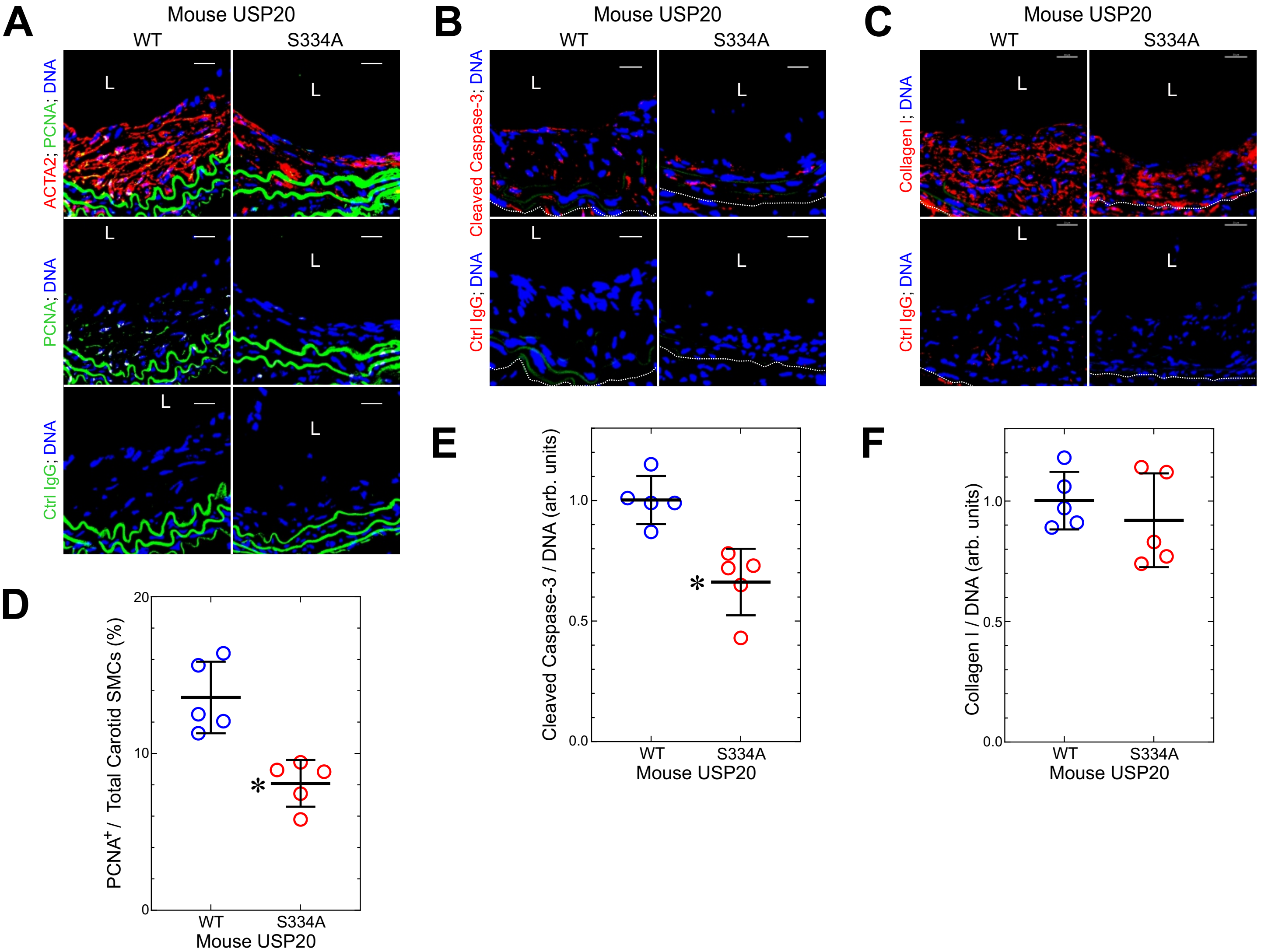

Figure S4. Phosphorylation of UPS20 on Ser334 augments arterial SMC proliferation and apoptosis. Carotid arteries of female mice from Fig. 2 were serially sectioned and immunostained with IgGs specific for PCNA, cleaved caspase-3, collagen I, or cognate isotype control ("Ctrl") IgG, and counterstained with Hoechst 33342 (DNA). Confocal fluorescence photomicrographs are shown; scale bars = 20 µm. "L" indicates lumen. *A*, PCNA-stained sections were co-stained for ACTA2 as described in Methods. Co-localization of green (PCNA) with either blue (DNA) or red (ACTA2) was performed using Imaris 9.2 software, to yield white or yellow, respectively. Elastic laminae demonstrate green autofluorescence. *B*, *C*, The dotted lines demarcate the external elastic laminae. *D*, Within the neointima and tunica media of each carotid artery cross section, the number of PCNA-positive cells was normalized to total number of cells; these values were plotted for 5 distinct carotid arteries per genetic group, along with means±SD. Compared with WT, *, $p < 0.01$ (unpaired *t* test). *E*, Within the neointima and tunica media of each carotid artery cross section, cleaved caspase-3 immunofluorescence was normalized to DNA fluorescence; these ratios were normalized to the mean value obtained for WT carotid arteries and plotted for 5 distinct carotid arteries per genetic group, along with means±SD. Compared with WT, *, $p < 0.003$ (unpaired *t* test with Welch's correction). *F*, Within the neointima and tunica media of each carotid artery cross section, collagen I immunofluorescence was normalized to DNA fluorescence; these ratios were normalized to the mean value obtained for WT carotid arteries and plotted for 5 distinct carotid arteries per genetic group, along with means±SD.

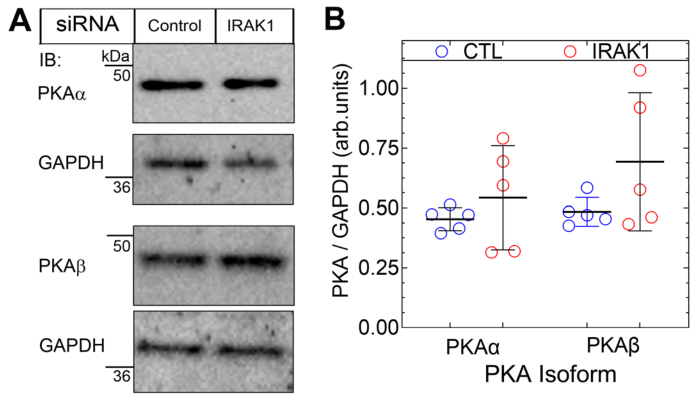

Figure S5. IRAK1 knockdown does not affect SMC PKA expression.
A. SMC lysates from experiments included in Figure 9C-E were immunoblotted for PKAα and PKAβ as indicated, and blots were reprobed for GAPDH. *B* PKAα and PKAβ band densities were normalized to cognate GAPDH band densities from 5 independent experiments, and ratios were plotted (with means ± SD) as arbitrary units.
